# Host-Microbiome Interaction and Cancer: Potential Application in Precision Medicine

**DOI:** 10.3389/fphys.2016.00606

**Published:** 2016-12-09

**Authors:** Alejandra V. Contreras, Benjamin Cocom-Chan, Georgina Hernandez-Montes, Tobias Portillo-Bobadilla, Osbaldo Resendis-Antonio

**Affiliations:** ^1^Instituto Nacional de Medicina GenómicaMexico City, Mexico; ^2^Human Systems Biology Laboratory, Instituto Nacional de Medicina GenómicaMexico City, Mexico; ^3^Coordinación de la Investigación Científica, Red de Apoyo a la Investigación-National Autonomous University of Mexico (UNAM)Mexico City, Mexico

**Keywords:** microbiome, cancer metabolism, systems integration, metabolome, next generation sequencing (NGS), precision medicine

## Abstract

It has been experimentally shown that host-microbial interaction plays a major role in shaping the wellness or disease of the human body. Microorganisms coexisting in human tissues provide a variety of benefits that contribute to proper functional activity in the host through the modulation of fundamental processes such as signal transduction, immunity and metabolism. The unbalance of this microbial profile, or dysbiosis, has been correlated with the genesis and evolution of complex diseases such as cancer. Although this latter disease has been thoroughly studied using different high-throughput (HT) technologies, its heterogeneous nature makes its understanding and proper treatment in patients a remaining challenge in clinical settings. Notably, given the outstanding role of host-microbiome interactions, the ecological interactions with microorganisms have become a new significant aspect in the systems that can contribute to the diagnosis and potential treatment of solid cancers. As a part of expanding precision medicine in the area of cancer research, efforts aimed at effective treatments for various kinds of cancer based on the knowledge of genetics, biology of the disease and host-microbiome interactions might improve the prediction of disease risk and implement potential microbiota-directed therapeutics. In this review, we present the state of the art of sequencing and metabolome technologies, computational methods and schemes in systems biology that have addressed recent breakthroughs of uncovering relationships or associations between microorganisms and cancer. Together, microbiome studies extend the horizon of new personalized treatments against cancer from the perspective of precision medicine through a synergistic strategy integrating clinical knowledge, HT data, bioinformatics, and systems biology.

## Introduction

Our body is integrated by a legion of microorganisms that coexist in all our tissues and, notably, with a symbiotic functional purpose. Furthermore, host-microbial interactions are beginning to be recognized for their outstanding influence on well-being or the emergence of diseases such as cancer. The advent of high-throughput (HT) technologies has allowed significant advancements in uncovering these correlations through the diversity and abundance of microorganisms in samples of normal and dysfunctional cohorts of human tissues associated with complex diseases such as obesity, type 2 diabetes, and cancer. For instance, in 2015, Mitra and co-workers reported the characterization of the microbiota at different stages of development of cervical intraepithelial neoplasia, and they observed a strong association between the severity of the disease and the vaginal microbiota diversity (Mitra et al., 2015). Furthermore, the association of the microbiota and obesity has also been explored, with observations of changes in the balance and relative abundances of Bacteroidetes and Firmicutes (Ley et al., 2006). Overall, these and other studies provide a glimpse of the central role that the microbiome has in a variety of biological processes in the human body such as in the regulation of fat storage, lipogenesis, fatty acid oxidation and energy balance (Gérard, 2016).

These findings that associate microbiome and phenotype dysfunctional states have contributed to a change in paradigms regarding the relationship between human body and microorganisms, and suggest elucidating the rules by which this interaction can confer wellness or disease. To this end, some challenges must be overcome. For instance, the development of new computational paradigms that contribute to the coherent interpretation of heterogeneous HT technologies, such as Next Generation Sequencing (NGS) and Metabolomics, and the construction of quantitative schemes capable of influencing clinical decisions in precision medicine.

In this review, we present the forefront of HT technologies and conceptual schemes in bioinformatics and systems biology for surveying the host-microbiome association and cancer progression. We expect that our review will be used as a technical and conceptual guide in human microbiome studies, present and discuss the advances in the field, and establish an introspective analysis of the next steps for linking microbiome studies and precision cancer medicine.

## Characterization of the microbiome using high-throughput technologies

The advent of HT technologies has positively impacted the elucidation of the metabolic and regulatory mechanisms by which hosts and microbes interact to determine a health or disease state in the host. In particular, NGS and techniques related to metabolome analysis such as mass spectrometry (MS) are valuable technologies for analyzing the microbiota composition and exploring the genetic, functional, and metabolic activity of the microbial community. Moreover, the use of these technologies enables us to explore the implications of the human microbiome to induce functional and dysfunctional states in a variety of human tissues. Here, we present the state of the art of these technologies and discuss some key findings to elucidate the relationship between the human microbiome and cancer.

### Next generation sequencing

Sanger sequencing, the first-generation of DNA sequencing technology developed by Frederick Sanger based on the selective incorporation of chain-terminating dideoxynucleotides by DNA polymerase, established the methodological principles for DNA sequencing (Sanger et al., 1977). The Sanger sequencing technique constituted the main part of the Human Genome Project in 2001 and was the principle for the first automatic sequencing machine (AB370) produced by Applied Biosystems (Liu et al., 2012). However, limitations in throughput and the high cost of Sanger DNA sequencing reduced the potential of sequencing for other applications, such as for the characterization of personal genomes and cancer whole-genome sequencing. In fact, the cost of the Human Genome Project was estimated to be approximately 1–3 billion dollars over a 15-year period (International Human Genome Sequencing Consortium, 2004). After 2004, when the International Human Genome Sequencing Consortium published the completed sequencing process of the human genome, different HT sequencing technologies emerged, promoting decreasing costs and increasing potential applications for human health (Reuter et al., 2015).

Through automated DNA sequencing instruments that use an attractive interaction among chemistry, engineering, software and molecular biology, dramatic improvements in sequencing technology have allowed revolutionary advances in our understanding of health and disease (Mardis, 2011, 2013). The launch of the Genome Sequencer system by 454 Life Sciences in 2005 highlighted the use of second-generation sequencing techniques employing massively parallel analysis. The second- and third-generation sequencing platforms, collectively known as NGS, are characterized by high data throughput, which can be used for a diverse range of scientific applications by changing the sample type and the manner of its preparation.

Many commercial second-generation sequencing platforms are now available, which follow a similar protocol: library/template preparation, clonal amplification and massively parallel sequencing. In terms of throughput per run, read length and accuracy, each platform has different specific features that make them useful for particular applications. Moreover, the newly emerged third-generation sequencing techniques, such as PacBio (Brown et al., 2014) and MinION (Quick et al., 2014), are performed on a single-molecule basis with no necessary initial DNA amplification step. These newer technologies can produce much longer reads compared with the second-generation sequencing platforms and have the potential to be less costly and less time-consuming.

Several reviews have covered these major platforms in high detail (Metzker, 2010; Mardis, 2013; Reuter et al., 2015). Of particular interest for this review is the application of NGS as an important tool that can provide detailed information about the taxonomic composition and the functional capabilities of the human microbiome for modern biomedical research. Some platforms are not discussed in this review, including Roche-454's pyrophosphate Genome Sequencer and ABI's SOLiD; instead, we attend to the platforms most commonly used today as technological tools in microbiome analysis as well as recent development (Table 1).

**Table 1:** **Comparison of next generation sequencing systems used in microbiome analysis**.

	**Illumina**	**Ion torrent**	**Pacific biosciences**	**Oxford nanopore**
Template preparation	Amplification of adaptor-ligated DNA fragments on a solid phase.	Amplification of adaptor-ligated DNA fragments by emulsion-PCR.	Ligation of a double-stranded region (the insert) onto a single-stranded hairpin loop on either end (SMRT-bell templates).	Ligation of DNA fragments to two adaptors; the first adaptor is bound with a motor enzyme and a molecular tether, and the second one is a hairpin oligonucleotide bound by a second protein (HP motor).
Sequencing chemistry	Sequencing by synthesis using reversible terminators.	Sequencing by synthesis coupled proton detection.	Sequencing by a strand displacing polymerase positioned in zero-mode waveguides (ZMWs) that incorporates phosphate-labeled nucleotides.	Sequencing by measuring electric current fluctuations when bases along the DNA strand translocate through a nanopore under an applied electric field.
Read length	MiSeq: Up to 300 bp NextSeq 500: Up to 150 bp HiSeq 2500: Up to 125 bp	Ion PGM System: 200- or 400-base reads Ion Proton System: Up to 200-base fragment reads Ion 5S: 200- or 400-base reads	PacBio RS II System: >20 kb	MinION: Median and maximum read lengths of ~6 and 65 kb, respectively
Throughput per run	MiSeq: Up to 13.2–15 Gb NextSeq 500: Up to 100–120 Gb HiSeq 2500: Up to 900–1 Tb	Ion PGM System: 600 Mb-1 Gb Ion Proton System: Up to 10 Gb Ion 5S: Up to 10–15 GB	PacBio RS II System: 500 Mb- 1 Gb	MinION: ~90 Mb
Advantages	The overall error rates are below 1%. Different sequencers optimized for a variety of throughputs.	The sequencing process does not require fluorescence and camera scanning, resulting in a fast method.	Direct sequencing of DNA without clonal amplification. Sequencing of the DNA molecule multiple times, increasing accuracy.	Direct sequencing of DNA without clonal amplification. Available device as USB-powered portable sequencer.
Limitation	The most common error is substitution.	The most common error types are insertions and deletions (indels). Homopolymer repeats longer than 6 bp lead increasing error rates.	The predominant errors are insertions (12%) and deletions (2%).	Error rates estimated for insertion, deletion and substitution are 4.9, 7.8, and 5.1%.
References	Dohm et al., 2008; Reuter et al., 2015	Rothberg et al., 2011; Liu et al., 2012	Travers et al., 2010; Carneiro et al., 2012	Quick et al., 2014; Ashton et al., 2015; Jain et al., 2015; Reuter et al., 2015

The appropriate selection of one platform depends on the particular aim and design of the study. Illumina's technology has had tremendous advances in output and reduction in costs over the last few years and, as a consequence, currently dominates the NGS market (Dohm et al., 2008; Reuter et al., 2015). Illumina's sequencing technology has been widely used in microbiome projects (Evans et al., 2014; Lambeth et al., 2015; Yasir et al., 2015), including the Human Microbiome Project (HMP Consortium, 2012a).

Although both the Illumina and Ion Torrent systems offer a number of advantages in terms of utility for generating usable sequences, its feature to obtain short read length makes them less suited for some particular scientific questions, including genome assembly, gene isoform detection, and methylation detection (Rothberg et al., 2011). Single-molecule real-time (SMRT) sequencing (third-generation sequencing platforms) offers an available approach to overcome these limitations. *De novo* genome assembly is one of the main applications of PacBio sequencing because long reads can provide large scaffolds (Travers et al., 2010; Carneiro et al., 2012; Rhoads and Au, 2015). In addition, using the direct sequencing protocol without library preparation offers the advantage of requiring a small quantity of DNA, just 1 ng for small genomes, over the other protocols that require 400–500 ng (Coupland et al., 2012). Moreover, SMRT sequencing methods can be used to study molecules other than DNA, for instance ribosomes (Uemura et al., 2010).

DNA sequencing using nanopore technology is another alternative method for producing long-read sequence data. The recent distribution of the MinION by Oxford Nanopore Technologies has made it possible to evaluate the utility of long-read sequencing using a device that resembles a USB memory stick (Ashton et al., 2015; Jain et al., 2015). Speed, single-base sensitivity and long read lengths make nanopore-based technology a promising method for HT sequencing. The MinION system has been used to sequence genomes of infectious agents, such as the influenza virus (Wang J. et al., 2015), to identify the position and structure of a bacterial antibiotic resistance island (Ashton et al., 2015), and as part of a genomic surveillance system of Ebola virus in which the sequencing process took as little as 16–60 min (Quick et al., 2016).

Rapid advances in sequencing technologies present widespread opportunities for microbiome studies using different platforms; however, the performance of the sequencing should be considered for the study design. Loman et al. reported that MiSeq had the highest throughput per run (1.6 Gb/run, 60 Mb/h) and the lowest error rates compared with 454 GS Junior or Ion Torrent PGM (Loman et al., 2012). In addition, Clooney et al. compared Illumina HiSeq, MiSeq and Ion PGM shotgun sequencing on six human stool samples, and found that optimal assembly values for the HiSeq were obtained for 10 million reads per sample, whereas the MiSeq and PGM sequencing depths were not sufficient to reach an optimal level of assembly (Clooney et al., 2016). Furthermore, MiSeq and PGM technologies provide a better functional categorization for predicting core genes from assembled contigs, possibly due to their longer read lengths (Clooney et al., 2016). Therefore, in some cases a combination of platforms could provide a more complete coverage of the studied genome.

The current sequencing assay protocols allow for two types of microbiome studies: (a) marker gene sequencing community identification, which surveys and counts microbes using amplicon sequencing of a single marker gene that is usually the 16S rRNA gene, and taxonomic assignment by bioinformatic methods; and (b) shotgun metagenomic sequencing, which surveys the entirety of all microbial DNA present in a sample using a collection of *ad-hoc* bioinformatic methods for gene and species identification purposes (Brown, 2015).

#### Amplicon sequencing

Classic microbiology methods are limited to the study of microbes that grow under specific sets of culture conditions; however, most microbial species are difficult or impossible to culture *in vitro*. For that reason, their full genetic spectrum was unknown until the advent of HT sequencing technologies, expanding our knowledge of the microbial world. The similarities and distinctions among bacterial species have become complex (Konstantinidis et al., 2006), so that, instead of a “species,” the term “operational taxonomic unit” (OTU) is used to characterize and infer the phylogenetic relationships between organisms grouped by sequence similarity (Blaxter et al., 2005; Koeppel and Wu, 2013; Schmidt et al., 2014). Usually, the 16S rRNA gene, which is a highly conserved gene in all prokaryotes, is amplified to analyze prokaryotic taxonomic composition in samples. However, this gene is approximately 1550 base pairs long making it difficult to sequence the whole gene through HT sequencing methods without an assembly step (Di Bella et al., 2013).

Instead of sequencing the entire 16S gene, one or more of its nine variable (V) regions are amplified using particular sets of primers. The choice of which variable region to use and amplify depends on factors related to the sample and experiment. For instance, evidence suggests that the V1–V3 region is better for taxonomical classification of species; however, some predictive studies show that the V3–V5 region results in a better classification of microbiota from disease vs. healthy specimens (Statnikov et al., 2013). Kim et al. analyzed different variable regions and recommended targeting of the V1–V3 and V4–V7 regions for the analysis of archaea and the V1–V3 and V1–V4 regions for the analysis of bacteria (Kim et al., 2011).

#### Shotgun metagenomic sequencing

Although the 16S rRNA is the most frequent gene used for studies of microbial community membership and structures, it has some limitations. The use of a particular set of primers for amplification of 16S and its PCR conditions can favor some taxa over others, creating bias in abundance counts (Statnikov et al., 2013). In addition, the 16S primers do not capture viruses and eukaryotes. Then, the shotgun metagenomic sequencing approach is commonly used to describe microbial communities without the biases inherent to PCR amplification of a single gene. In principle, shotgun sequencing provides robust estimates to identify the whole genomes present in a biological sample, including genome sequences of viruses and other functional DNA elements (Brown, 2015).

Metagenomic analysis is much more challenging than amplicon sequencing due to the consideration of whole genomes instead of a particular gene. Indeed, hundreds of millions of reads must be generated and analyzed for each sample, taking advantage of very deep sequencing on the Illumina HiSeq or similar instruments. In addition to shotgun metagenomic analysis, metatranscriptomic analysis using direct cDNA sequencing, which is known as RNA sequencing (RNA-seq), allows for the analysis of all of the RNA of a sample to determine which genes are transcribed and for monitoring gene regulation over time, which is particularly interesting when studying changes in the microbiota in response to perturbations (Valles-Colomer et al., 2016).

Due to technical difficulties such as isolation of high quality RNA from biological samples or the presence of mRNA from the host, the application of RNA-seq to the study of the human microbiota in cancer is still limited. To date, a couple of interesting studies related to the metatranscriptome and the microbiome have been published. In 2014, Franzosa and coworkers reported the correlation between the metagenome and metatranscriptome of the healthy human gut microbiome. These findings showed that 41% of microbial transcripts are in concordance with their genomic abundances, while sporulation and some pathways of amino acid biosynthesis are underexpressed, and methanogenesis and ribosome biogenesis are up regulated. Interestingly the subject-specific metatranscriptomic variation was more significant than the metagenomic variation (Franzosa et al., 2014). In 2015, Versluis and coworkers explored the gut metatranscriptomes for the expression of antibiotic resistance genes. Their results showed that resistance gene expression could be constitutive or could have different roles other than antibiotic resistance (Versluis et al., 2015).

**Figure 1:**
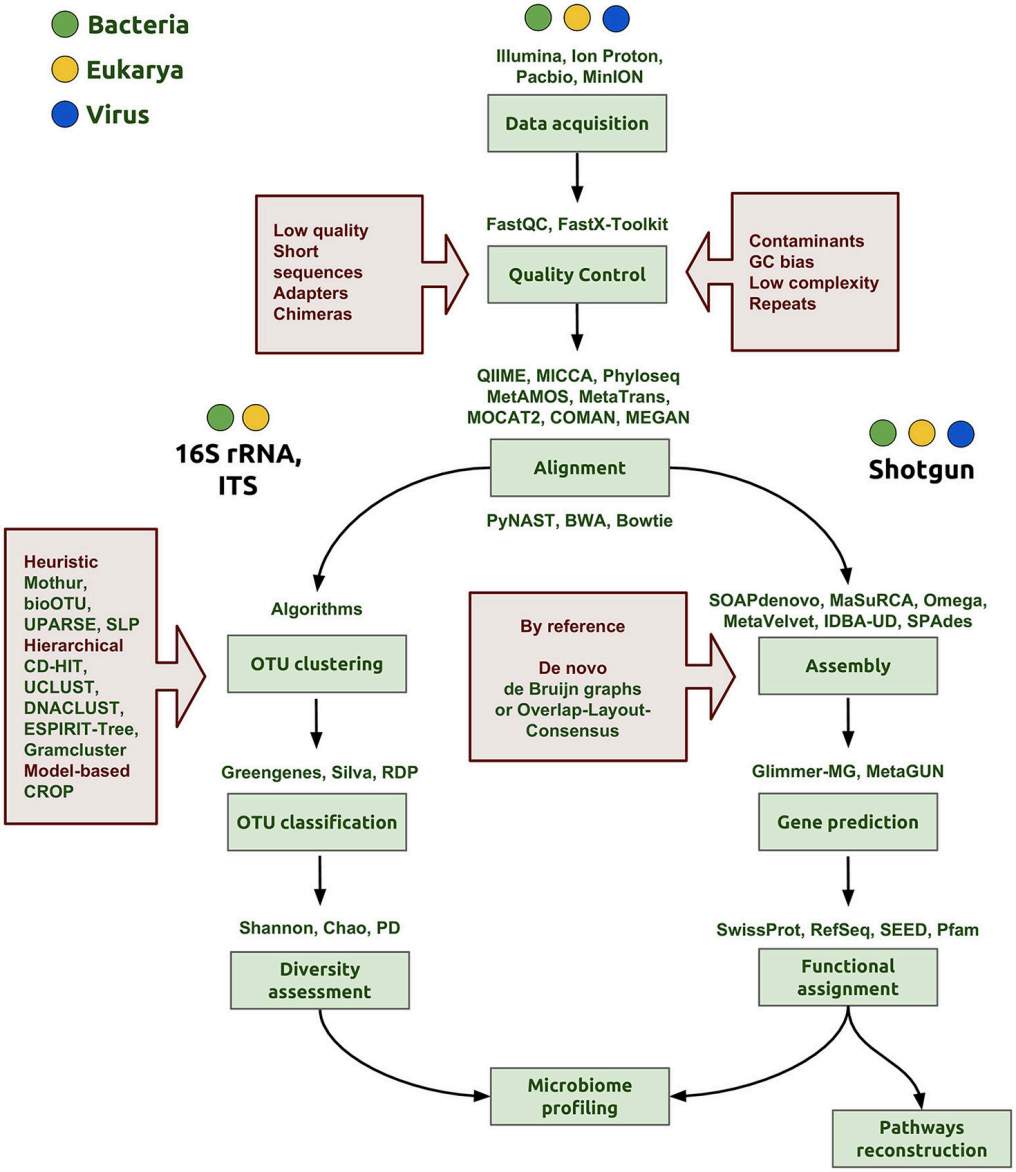
**Bioinformatics workflow of microbiome profiling**. The first step is the data acquisition that can be derived from any NGS technology (Illumina, IonProton, PacBio) and generating of the FASTQ file to proceed with the analysis. In the quality control step, the aim is to clean and eliminate possible errors in data, for example, to discard low quality score and very short reads, quimeric and adapter sequences. In addition, it is important to evaluate the presence of some contaminants from other organisms, specific GC content bias or repeated sequences that may interfere with the assembly step. The following steps depend on the nature of data, whether the aim is to sequence a marker gene, such as the 16S rRNA gene or ITS, or to perform shotgun metagenomic sequencing. OTU clustering is a critical step and many algorithms and strategies have emerged to accomplish a proper classification of sequences for a more accurate determination of taxa proportions and diversity indexes (diversity assessment). Good assemblies and alignments are an important aspect to reach correct gene predictions in the whole genome pipeline. In the functional assignment step, we gather a biological understanding for regulation and gene pathway reconstruction, obtaining finally the microbiome profiling.

After sequence data have been obtained, the next step in the NGS pipeline is the bioinformatics analysis of the reads, which include quality control, assembly and, finally, microbiome profiling (Figure 1). In each step of the bioinformatic pipeline, there are diverse computational methods that can be applied based on the organisms, the biological question being explored, and the technology applied to the samples. There are three initial steps in common when the 16S rRNA gene is used for prokaryotes, the nuclear ribosomal internal transcribed spacer region (ITS) for fungi or shotgun sequencing: (1) data acquisition or generation of FASTQ files (a common format for sharing sequencing read data); (2) quality control; and (3) assembly of the reads (Figure 1).

#### Data acquisition

The NGS methodologies provide data files in different formats depending on the platform used. For instance, the Illumina platform generates ^*^.bcl binary files containing base call and quality for each tile in each cycle, while Oxford Nanopore Technologies provide the data in binary files in HDF5/FAST5 format, which contains a number of hierarchical groups, datasets and attributes (Watson et al., 2014). However, to proceed with the analysis, both data files need to be converted to FASTQ format. The FASTQ files have four lines per sequence: sequence identifier, raw sequence, quality score identifier and quality scores encoded in Phred format. Phred quality scores are a measure associated with the assurance of each nucleotide in the sequence.

#### Quality control

Routinely, before starting a data analysis, a primary sequence analysis should be performed, where various data parameters are evaluated such as the quality scores of the sequences, global CG content, and the repeat abundance and the proportion of duplicated reads. The main tool to perform this is the FastQC (http://www.bioinformatics.babraham.ac.uk/projects/fastqc/) or the FASTX-Toolkit, which is a collection of command line tools. Parameters for good quality data include a Phred quality score above 28, low percentage of duplicated sequences, no adapter content, and GC count per read close to the theoretical distribution. Another useful tool for quality assessment and processing of HT DNA sequence data is the Bioconductor's package ShortRead (Morgan et al., 2009).

#### Assembly

The assembly processing of contigs consists of searching for overlapping reads, alignment and merging sequences to reconstruct the entire original sequence. There are two main approaches for genome assembly: *de novo* and reference guide. In the novo assembly approach, there are currently two main methods: Overlap-Layout-Consensus (OLC) and De Bruijn Graph (BG). OLC methods are based on overlap graphs, and their process has three steps: (1) searching for overlapping reads comparing all-against-all, (2) construction and manipulation of an overlap graph leading to an approximate read layout, and (3) constructing the consensus sequence using multiple sequence alignments (Miller et al., 2010). The BG method involves the definition and alignment of K-mers, where the K parameter denotes the length in bases of these sequences; the overlap is between k-mers, not between reads.

In the context of obtaining the microbiome profile of a sample using the 16S rRNA gene, three phases can be distinguished: OTU clustering, OTU classification and diversity assessment. The metrics for microbiota description include species richness and phylogenetic diversity, distance matrices of samples, alfa and beta diversity, rank abundance distributions and statistical analysis of ordering and classification. OTU clustering is a key step for *de novo* OTU construction that has an important efect on the estimation of species abundance and diversity. There are some recent comparisons of several of these clustering methods (Chen et al., 2013; Kopylova et al., 2016). Alternatively, to the direct construction of OTU clusters, more recently, DADA2 addresses the sequencing errors and its correction to properly identify the sequence variants at the strain level (Callahan et al., 2016). Further taxonomic assignment to the sequence table can be accomplished via Greengenes (DeSantis et al., 2006), SILVA (Quast et al., 2013) or a dedicated human intestinal 16S database (Ritari et al., 2015). There are different software options to analyze this kind of data from end to end such as QIIME or Mothur (Schloss et al., 2009; Caporaso et al., 2010; Navas-Molina et al., 2013), MICCA (Albanese et al., 2015) or phyloseq developed in R language (Mcmurdie and Holmes, 2013; Heazlewood et al., 2015).

While amplification of the 16S rRNA gene is performed to determine the diversity of and quantify the abundance of bacteria, metagenomic shotgun sequencing aims to recover genomes (Smits et al., 2015), describe the genomic structure and survey the metabolic capabilities of the different microorganism in a community. The most common strategy to reconstruct genomes and recover global functional pathways from metagenomic data from reads involves: (1) gene prediction, (2) functional assignment, and (3) pathway reconstruction (Abubucker et al., 2012).

Accurate gene prediction is critical for functional assignment. With the intent of increasing the accuracy of prediction, some authors recommend using algorithms that take into account significant differences between coding and non-coding sequences to identify open reading frames, di-codons frequency, GC content of coding sequences, preference bias in codon usage and patterns in the use of start and stop codons (Escobar-Zepeda et al., 2015).

From a practical point of view, there are several packages and suites to perform metagenomic analysis taking into account a variety of statistical tools (Supplementary Table 1). For instance, MetaGeneMark uses direct polynomial and logistic approximations of oligonucleotide frequencies, and it evaluates the dependencies between the frequencies of oligonucleotides with different lengths and the GC% of a nucleotide sequence (Zhu et al., 2010); Glimmer-MG, which is based on Glimmer, uses the interpolated Markov models with variable-order for capturing sequence compositions of protein-coding genes (Kelley et al., 2012); FragGeneScan incorporates sequencing error models and codon usages in a hidden Markov model to predict ORFs in short reads (Rho et al., 2010); and Orphelia is a gene finder based on the machine learning approach (Hoff et al., 2008).

A common strategy in metagenomics pipeline is the partitioning or clustering of reads (for example, for the exclusion of rRNA, tRNA or other specific DNA) by alignment methods (Kopylova et al., 2012; Wood and Salzberg, 2014). This allows taxonomy assignment and classification of reads. Improvements in terms of speed and accuracy of these tasks have been reached by various methods implemented in Phymm and PhymmBL (Brady and Salzberg, 2009), LMAT (Ames et al., 2013), mOTUs (Sunagawa et al., 2013), and more recently Kraken (Wood and Salzberg, 2014), MetaPhlAn2 (Truong et al., 2015), and SMART (Lee et al., 2016). For a better estimation of gene abundances, methods that uses a machine learning approach, such as MUSiCC (Manor et al., 2015). All these methods rely on a reduced database search of single copy genes, wide coverage phylogenetic markers or hidden Markov models using training sets. Others use combined methods of genomic signatures, marker genes and optional contig coverages (Lin and Liao, 2016). Peabody and coworkers present a recent comprehensive evaluation of metagenomic classification methods (Peabody et al., 2015).

Functional assignment is performed on the predicted open reading frame or predicted proteins by sequence similarity search to well-cured databases, using tools such as BLAST (local alignments), FASTA (global alignment) or HMMER (hidden model Markov profiles) when sequence identity is low. These analyses can be performed using locally installed software; alternatively, for users with no bioinformatic training, there are different suites for analysis, such as MG-RAST (Wilke et al., 2016), IMG/M (Markowitz et al., 2012; Wilke et al., 2016), JCVI and Metagenomics Reports (METAREP) (Goll et al., 2010; Markowitz et al., 2012; Wilke et al., 2016) or MEGAN (Huson and Weber, 2013), MetAMOS (Treangen et al., 2013), MOCAT2 (Kultima et al., 2016), and MetaTrans (Martinez et al., 2016) which are software designed to simplify all metagenomics or metatranscriptomics pipeline; preprocessing, assembly, annotation and analysis.

Having obtained a high quality functional annotation, the process of metabolic pathway reconstruction is extremely useful to identify, at a systemic level, those pathways with a primary role in supporting the phenotype. For mapping each gene in a metabolic pathway and analyzing missing enzymes (due to an analogous enzyme that is performing the same function), two different databases can be used: KEGG (Ogata et al., 1999) and MetaCyc (Karp, 2002). For instance, KEGG has implemented GhostKOALA as a tool for metagenomic analysis, which is based on a non-redundant dataset of pangenome sequences (Kanehisa et al., 2016).

### Metabolomics

Host-microbiome interactions encompass an exchange of metabolites and signaling molecules, some of them with an essential role to establish a proper functionality in the host and the microbial community. This crosstalk depends on a variety of factors such as the microbiome composition and external ambiances. Understanding the metabolic activity of these communities and how impacts the host has been the focus of many studies. Some of them associating metabolic biomarkers with the development of disease.

With the aim of disentangling this complex metabolic communication and surveying the metabolic pathways that actively participate in the community, metabolomics–embracing the massive quantitative measurement of intracellular or extracellular metabolites in biological samples such as human stool (Weir et al., 2013)–has been established as the more suitable HT technology to characterize the phenotype and dynamic response of living systems (Nicholson and Lindon, 2008; Marcobal et al., 2013; Diener et al., 2016).

**Figure 2:**
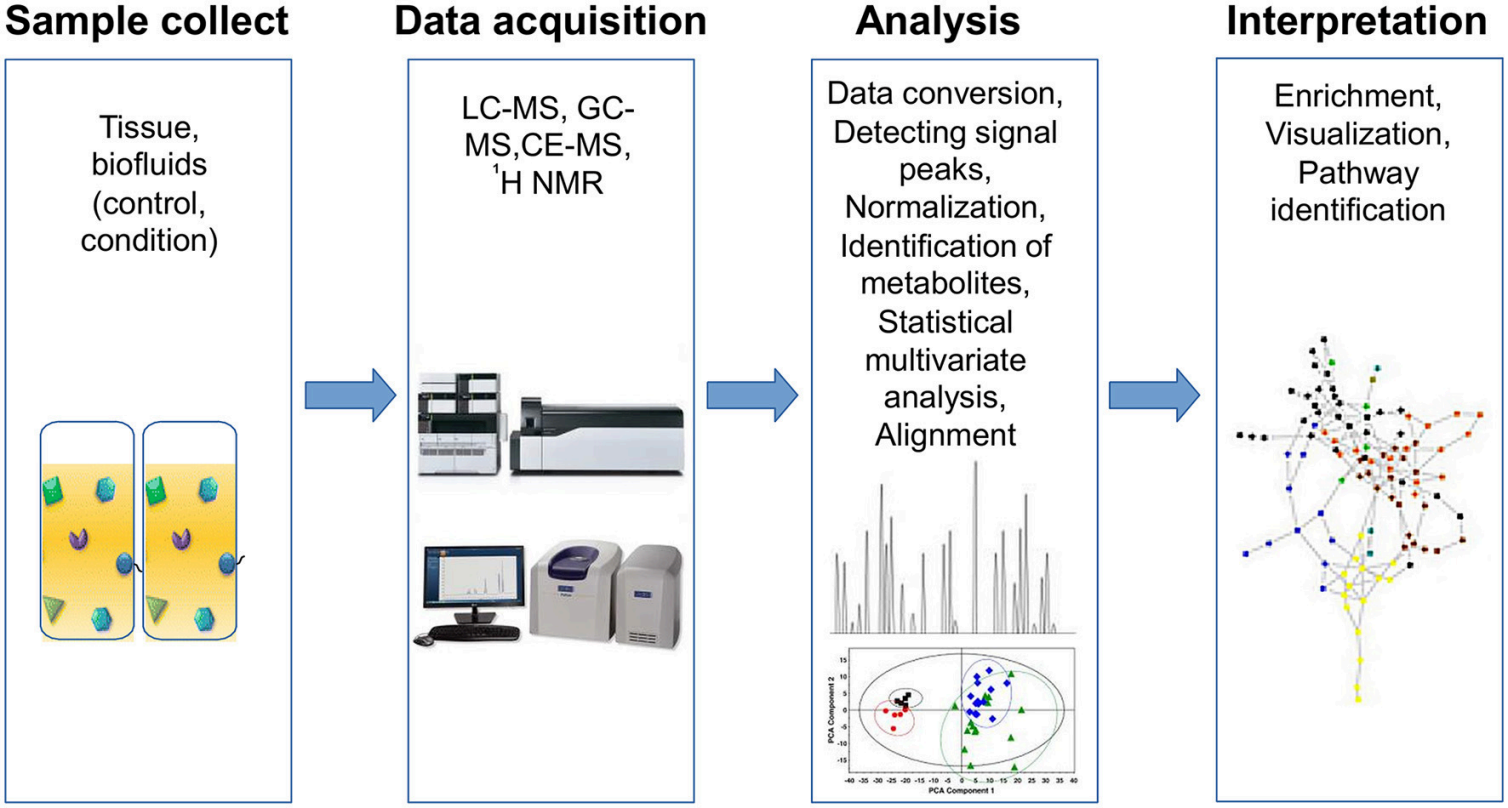
**Workflow for metabolomics analysis**. Metabolomic studies involve four general steps: (1) sample collection method, which depends on the type of tissue and must consider the type of storage, preservation and preparation of each sample, (2) data acquisition, involves sample analysis and quality control, (3) analysis data, includes normalization and identification of metabolites using specialized software for statistical analysis, and (4) data interpretation, which must be integrated and modeled to raise new hypotheses.

Metabolomic studies can be performed by using three basic approaches: (1) fingerprinting or endo-metabolome, searching for metabolites within the organisms under study; (2) footprinting or exo-metabolome, analyzing metabolites from the environment around the organism under study; and (3) metabolome profiling, where the goal is to screen one or more specific compounds (Patel et al., 2015). A typical metabolic study has four basic steps: sample collection, data acquisition, bioinformatic analyses and biological interpretation (Briefly described in Figure 2).

Currently, two main technologies are used in metabolomics; MS and nuclear magnetic resonance spectroscopy (NMR). MS is a highly sensitive method for detection, quantification and structure elucidation of hundreds of metabolites. Given the wide spectrum of molecular weights of metabolites in samples, it is necessary to separate metabolites to improve the sensitivity and accuracy of detection. Thus, MS is often coupled with different separation techniques such as gas chromatography (GC-MS), liquid chromatography (LC-MS) and capillary electrophoresis (CE-MS) (Gowda and Djukovic, 2014). All of these techniques have been used for clinical studies, and each has advantages and limitations. For instance, GC-MS has high-resolution capability, but it requires volatile compounds or compounds made volatile by chemical derivatization. LC-MS is a very sensitive technique, and it has the advantage of not requiring chemical derivatization of compounds; however, it has poor resolution. Also, the high capacity of CE-MS to separate compounds allows its use as a platform for multiplexing samples (Johanningsmeier et al., 2014; Nagana Gowda and Raftery, 2015).

On the other hand, NMR spectroscopy is a technique with high reproducibility and is able to absolutely quantify metabolites using a single reference; because it is a non-destructive technique, the samples can be used for re-analyses using other methods (Nagana Gowda and Raftery, 2015). NMR spectroscopy has two variations: ^1^H-NMR and high-resolution magic angle spinning NMR (HR-MAS-NMR).

After analyzing samples, it is necessary to interpret the data. Common analysis procedures involves data conversion, detecting signal peaks, alignment (i.e., comparison between different datasets to eliminate migration times shifts) (Katajamaa and Orešič, 2007), normalization and identification of metabolites. Processed data requires multivariate statistical analysis to find samples or variables accounting most of the variability between datasets and potential biological roles; therefore, methods such as partial least square discriminant analysis (PLS-DA), principal component analysis (PCA), hierarchical clustering analysis (HCA) and orthogonal partial least square discriminant analysis (OPLS-DA) are widely used. A number of free software packages and databases for metabolic analysis are available, and these are summarized in Supplementary Table 1. Visualizing tools can leverage the interpretation of results, both heatmaps and pathways are widely used to perform this task (Supplementary Table 1).

Finally, it is important to standardized data to share it in public databases, this could facilitate experimental replication between laboratories and maximize the value of metabolomic data (Fiehn et al., 2006). Additionally, the Human Metabolome Database (HMDB) is a metabolome project, analogous to the Human Genome Project, which aims to provide a comprehensive database of detected and biologically expected human metabolites. Currently, the HMDB has more than 40,000 metabolite entries (Wishart et al., 2012). The enrichment of these valuable tools can provide a better understanding of the characteristics of health and disease states when combined with other clinical and modeling approaches to fill the gap between the genotype and phenotype relationship (Diener et al., 2016).

To study the metabolic changes in health and disease, we can analyze the metabolites produced solely by the host, those produced or modified by the microbiome, or the metabolites jointly contributed from host-microbiome interactions (Guo et al., 2015). In cancer metabolomic research, there are different types of samples to study, including fluids such as urine, blood, saliva, breath condensate, cerebrospinal fluid, and pancreatic juices or tissue, and in each case require of particular method for storing and preparing the sample for processing (Spratlin et al., 2009). Additionally, metabolomics can help us to track those metabolites found in our environment that can influence the phenotype, such as diet, chemical exposure, xenobiotics, supplements or drugs (de Raad et al., 2016). Here, we briefly review some studies related to cancer metabolomics and host-microbiome co-metabolism.

Cancer cells have a specific metabolic demand to proliferate, increase their growth and sustain their malignant phenotype (Resendis-Antonio et al., 2015). Notably, this physiological state is represented by changes in the metabolic profile of human tissue. The identification of these metabolic alterations is a crucial point to define the phenotype, design new therapeutic targets and explore the evolution of the disease (Locasale et al., 2009; Yun et al., 2009; Ramirez et al., 2013).

Metabolomic studies have led us to search for new biomarkers in cancer, and these findings have had important implications for surveying the mechanisms of a variety of cancers such as bladder (Rodrigues et al., 2016), breast (Jobard et al., 2014), pancreatic (Di Gangi et al., 2015), gastroesophageal (Abbassi-Ghadi et al., 2013), gastric (Abbassi-Ghadi et al., 2013; Chan et al., 2016), and oral (Mikkonen et al., 2015) cancer. For instance, in the case of gastric cancer, three potential biomarkers, 2-hydroxyisobutyrate, 3-indoxylsulfate and alanine, were identified in urine samples using ^1^H-NMR spectroscopy. Revealing that those patients have a particular metabolic profile (Chan et al., 2016).

Other more comprehensive approaches involve the study of microbiome metabolites and their interactions with the host, i.e., synthesis, absorption, and potential physiological effects on the host. There are several studies that have been able to discern the different metabolites in the human gut microbiome and their relationships with health and disease (Sharon et al., 2014). Additionally, there are *in vivo* studies observing the effects of the human gut microbiota on the metabolism of biofluids of humanized mice (Marcobal et al., 2013; Smirnov et al., 2016). By characterizing, discerning and associating metabolite levels with genetics and external factors such as diet and the microbiome, metabolomics can aid in diagnostics and expand the clinical scope toward the realization of precision medicine (Beebe and Kennedy, 2016).

For instance, Guo et al. analyzed the plasma metabolites from healthy volunteers, identifying 600 metabolites covering 72 biochemical pathways, ranging from biosynthesis, catabolism, gut microbiome activities, and xenobiotic metabolism. Also, the metabolome profiles were associated with whole-exome sequencing and clinical records to identify metabolic abnormalities associated with disease (Guo et al., 2015). This approach exemplifies how complementing genetic and metabolic analysis can help to improve diagnosis and medical interventions such as dietary changes, evaluate drug response and the discovery of biomarkers.

## Elucidating the host-microbiome interactions and cancer development

In the emergence of complex diseases such as cancer, the relationship between the environmental influence, the microbiome and cancer appearance can be very entangled. The body offers a suitable and nutrient-rich microenvironment to resident microbes, while the microbiome assists humans in metabolic or immune tasks. Additionally, the microbiota provides humans with non-nutrient essential factors, such as vitamins, and impedes pathogens from establishing (Zitvogel et al., 2015). Differences in microbial and possibly viral compositions between healthy subjects and those affected by diseases have been identified (Blumberg and Powrie, 2012; Koeth et al., 2013; Bultman and Jobin, 2014; Clavel et al., 2014; Tilg and Moschen, 2014). Broadly defined, this imbalance, referred as dysbiosis, imply deviations in the composition of resident commensal communities from the ones found in healthy individuals (Petersen and Round, 2014).

Most of the current research exploring the effects of host-microbe interplay in cancer is focused on colorectal cancer (CRC). By using genomic approaches, some studies have compared the mucosal surface and the intestinal lumen microbiota between healthy patients and those with CRC (Chen et al., 2012; Kostic et al., 2012; Sanapareddy et al., 2012). Although there is no consensus between studies, some taxa are associated with a protective function (e.g., *Roseburia*) while others are associated with potentially detrimental effects (e.g., *Fusobacterium, Klebsiella*, and *Escherichia/Shigella*) (Jobin, 2013; Thomas and Jobin, 2015). This suggests a dysbiotic or differential community composition correlated with CRC development. However, among the open issues about host-microbiome interactions in disease, we ignore the role of the microbiome as a driver or consequence of cancer development (Tjalsma et al., 2012).

Altered cellular metabolism and inflammation are proposed host dependent hallmarks of cancer (Hanahan and Weinberg, 2011). Even when host-microbiome interactions might not be considered essential for cancer appearance, or its effects are indirect, some cancers, such as CRC, might have an important microbial component. *In vitro* studies have reported a signaling process between bacterial quorum-sensing peptides (QSPs) and cancer cells. *Bacillus* derived QSPs are synthesized when there are bacterial stressors and are able to induce tumor cell invasiveness in a process called epithelial-mesenchymal-like (EMT-like) process (involved in CRC metastasis) (Wynendaele et al., 2015). The QSPs contributed both to metastatic and angiogenesis behaviors under these settings (De Spiegeleer et al., 2015; Wynendaele et al., 2015). Furthermore, in other kinds of cancer, the result of microbial activities can reduce the effectiveness of chemotherapy (Wallace et al., 2010) or influence the development of tumors distant from the gut (Iida et al., 2013).

**Figure 3:**
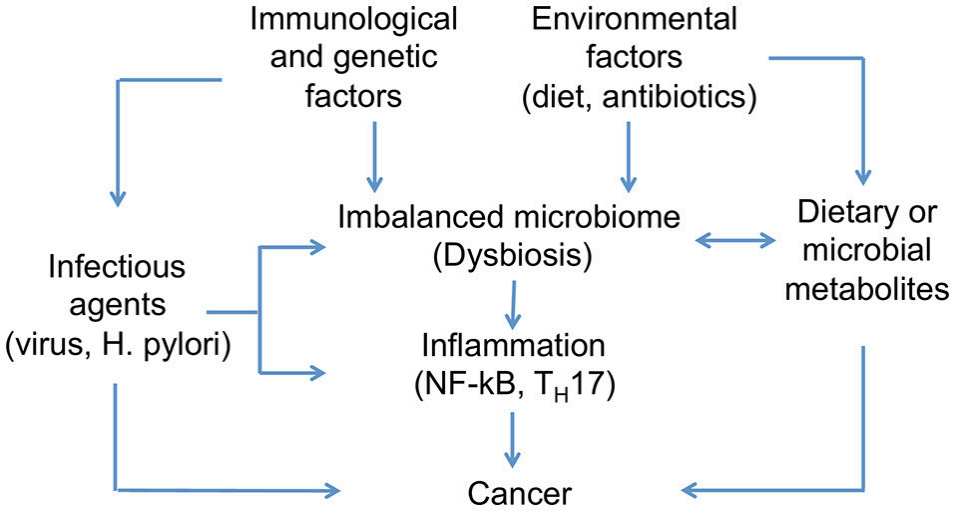
**Host-microbiome interactions implicated in cancer development**. Differences in microbial composition between healthy individuals and those affected by cancer have been identified. Genetic and environmental factors can disrupt the healthy condition of human microbiome and promote microbial dysbiosis. Infectious agents are one of the main contributors to dysbiosis and cancer development, in addition to diet, which has been recognized as one of the major players in determining microbiome composition. Moreover, microbes associated to cancer appear to activate pro-inflammatory pathways on host tissues.

Genetic and environmental factors disrupting the healthy relationship between hosts and microbiomes can provoque dysbiosis and promote cancer development (Figure 3). Lifestyle, diet, and early exposure have been recognized as major players in determining the microbiome composition. Additionally, different metabolites produced by the intestinal microbiota are proposed to play both cancer-promoting and cancer-protecting roles; however, factors determining different outcomes are not completely understood (Bultman and Jobin, 2014). Characterizing bacterial OTUs consistently altered across studies, and attributing to them the presence of specific diseases can be difficult given the inter-individual variations (Zackular et al., 2013). This suggests the need to understand what are the possible roles of the microbiome in this process. In this regard, we will review three major factors that can promote microbial dysbiosis and cancer development: (1) infectious agents, (2) diet- and microbial-derived metabolites; and (3) inflammatory mediators.

### Infectious agents in cancer

Infectious agents are one of the main contributors to cancer development. The linkage of infection with some biological agents and carcinogenesis in humans started more than a century ago when Francis Peyton Rous began his famous cancer virus transmission experiments at the Rockefeller Institute, USA (Moore and Chang, 2010). Eleven biological agents have been identified as group 1 carcinogens by the International Agency for Research on Cancer (IARC) (Bouvard et al., 2009). These include Epstein-Barr virus (EBV), hepatitis B and C viruses (HBV and HCV, respectively) Kaposi sarcoma herpesvirus (KSHV, also known as human herpesvirus type 8, HHV-8), human immunodeficiency virus type 1 (HIV-1), human papillomavirus (HPV) type 16 (HPV-16), human T-cell lymphotropic virus type 1 (HTLV-1), *Helicobacter pylori* (*H. pylori*), *Clonorchis sinensis* (*C. sinensis*), *Opisthorchis viverrini* (O. *viverrini*), and *Schistosoma haemotobium* (*S. haemotobium*). Although HIV does not directly cause cancer, its infection strongly increases the incidence of many different human cancers. Among these cancers, those associated with the herpesviruses KSHV and EBV are the most strongly enhanced by immunosuppression (Bouvard et al., 2009).

Specific infections represent major cancer risk factors with an estimated 2.1 million (16.4%) of the 12.7 million new cases in 2008 attributable to infection. This fraction is substantially higher in less developed regions of the world (23.4% of all cancers) than in more developed regions (7.5%). The most important infectious agents are *H. pylori*, hepatitis B and C viruses and HPV, which together are responsible for 1.9 million cases of gastric, liver and cervix uteri cancers, respectively (de Martel et al., 2012). A better understanding of the role of infectious agents in the etiology of cancer is an essential element for precision medicine, because such cancers are theoretically preventable by proper vaccination or early treatment of infection (IARC Working Group on the Evaluation of Carcinogenic Risks to Humans, 2012).

The IARC estimates that one in five cancer cases worldwide are caused by infection, with most being caused by viruses (Bouvard et al., 2009). The first human tumor virus, Epstein-Barr virus, also known as human herpesvirus 4 (HHV-4), was described in 1964 in cell lines from African patients with Burkitt's lymphoma (Epstein et al., 1964). EBV is invariably associated with the non-keratinizing type of nasopharyngeal carcinoma (NPC), which represents 80% of NPC cases, and new evidence points to a role for EBV in 5–10% of gastric carcinomas. EBV infection is observed to occur mostly in the upper middle portions of the stomach rather than in the lower part of the stomach (Shah and Young, 2009).

Chronic infection with Hepatitis B virus (HBV) and hepatitis C virus (HCV) is known to cause hepatocellular carcinoma (Song et al., 2016). Several epidemiological studies suggest that HCV may be involved in the pathogenesis of several B-cell lymphoproliferative disorders. In particular, sufficient evidence is available to indicate that chronic infection with HCV can also cause non-Hodgkin lymphoma (Hermine et al., 2002). Evidence of HTLV-1 infection was initially found in at least 90% of adult T-cell leukemia and lymphoma (ATLL) cases; subsequently, HTLV-1 infection became part of the diagnostic criteria for ATLL (Oh and Weiderpass, 2014). KHSV is a causal factor for Kaposi sarcoma and, more recently, MCV, a novel member of the polyomavirus family, has been identified. There is some evidence that MCV has an important role in the development of Merkel cell carcinoma, a rare skin cancer arising in elderly and chronically immunosuppressed individuals (Shuda et al., 2008).

It is very well established that infection with specific types of HPV can cause cervical cancer. Global epidemiological studies identified HPV 16, 18 and a few others as major risk factors for cervical cancer (zur Hausen, 2009). In addition, there is strong epidemiological evidence for the involvement of HPV infection in the carcinomas of the cervix, penis, vulva, vagina, anus, upper aerodigestive tract, and head and neck. The majority of HPV-related head and neck cancers are located in the oropharynx (Hettmann et al., 2015). Multiple meta-analyses support the discovery of a higher HPV detection rate in regions associated with high risk for esophageal squamous cell carcinoma (ESCC), compared to low-risk areas. Additionally, a potential role of HPV in the rise of esophageal adenocarcinoma (EAC) was proposed recently; however, future studies are required (Xu et al., 2015).

The prevalence of *H. pylori* infection varies widely by geographic area, age and socioeconomic status. In less developed regions, it may reach 80%, while, in more developed regions, the prevalence is 40% or less (Brown, 2000). *H. pylori* infection is limited to the distal part of the stomach, and chronic infection is associated with non-cardia gastric carcinoma. *H. pylori* yields various virulence factors that may dysregulate host intracellular signaling pathways, controlling the immune response associated with the induction of carcinogenesis. Of all virulence factors, *cag*A (cytotoxin-associated gene A), and its pathogenicity island (*cag* PAI), and *vac*A (vacuolating cytotoxin A) are the major pathogenic factors (Ahn and Lee, 2015). *H. pylori* can modulate the immune response through activating growth factors and cytokines (Amedei et al., 2009). For instance, the *H. pylori* secreted peptidyl prolyl cis, trans-isomerase, HP0175, is one of bacterial antigens recognized by sera of *H. pylori* infected patients, that is able to activate both epidermal growth factor receptor and NF-κB pathway, and drives gastric T helper 17 (T_H_17) responses in patients with distal gastric adenocarcinoma (Amedei et al., 2014).

Regarding helminth infections, chronic infections with the liver flukes *C. sinensis* and *O. viverrini* are associated with cholangiocarcinoma. Liver fluke antigens stimulate both inflammatory and hyperplastic changes in the infected bile ducts, which undergo severe pathological transformations. The relative risk for this adenocarcinoma is estimated to be 7.8 for individuals infected with *O. viverrini* and 7.7 for those infected with *C. sinensis* (IARC Working Group on the Evaluation of Carcinogenic Risks to Humans, 2012). Approximately 5–10% of cholangiocarcinoma is caused by chronic *C. sinensis* infection in endemic areas, which are located in China, Korea, Thailand, Laos, Vietnam, and Cambodia (Oh and Weiderpass, 2014). On the other hand, *S. haematobium* is a parasitic flatworm associated with bladder cancer that infects millions of people, mostly in the developing world. In *in vitro* models exposed to total antigens Botelho et. al. found increased cell proliferation, decreased apoptosis, up-regulation of the anti-apoptotic molecule Bcl-2, down-regulation of the tumor suppressor protein p27, and increased cell migration and invasion (Botelho et al., 2010).

Infectious agents can be direct carcinogens, such as the HTLV-1 and the KSHV, which express viral oncogenes that directly contribute to cancer cell transformation, or indirect carcinogens by causing chronic inflammation, which eventually leads to carcinogenic mutations in host cells, such as *H. pylori*, the major cause of gastric carcinogenesis. In addition, carcinogenesis would result from the interaction of multiple risk factors including those related to the infectious agent itself (virulence factors, variants, or subtypes), host-related factors (gene polymorphisms and immune system status) and environmental aspects (smoking, chemicals, ionizing radiation, immunosuppressive drugs, or another infection that may lead to reactivation of latent oncogenic viruses such as EBV or KSHV) (IARC Working Group on the Evaluation of Carcinogenic Risks to Humans, 2012). Further studies should be conducted to elucidate in detail the contribution of these additional factors to the development of cancers associated with infectious agents.

### Diet and microbial-derived metabolites in cancer

Microbiome-derived metabolites are gaining recognition for their potential participation in cancer development (Louis et al., 2014). Clearly, diet is a major source for the production of those metabolites and has to be taken into account along with microbiome composition and activities. For example, high fat and high protein consumption is characteristic of modern western diets (Hughes et al., 2000; Albenberg and Wu, 2014), and this particular dietary composition is currently recognized as a risk factor for cancer occurrence (Bouvard et al., 2015; Gallagher and LeRoith, 2015). In this section, we will present some example *in vitro* and *in vivo* studies of microbiome-derived metabolites related to cancer development, and explore its possible application as biomarkers.

#### Secondary bile acids

In the liver, enzymatic oxidation of cholesterol generates bile acids (BA) that function as detergents that facilitates digestion and absorption of lipids; while also acting as signaling molecules related to metabolic homeostasis (de Aguiar Vallim et al., 2013). The presence BAs in the colon promotes its subsequent conversion to secondary bile acids (SBA) by means of bacterial enzymes. Species with 7-α-dehydroxylating enzymes, can convert the host's BA into SBA (Ou et al., 2013) and those can act as carcinogens (Bernstein et al., 2005).

*In vitro* studies have shown that 1-h exposure to SBAs Deoxycholic Acid (DCA) or Lithocholic Acid (LCA) causes extensive DNA damage at physiological concentrations in a dose-dependent manner (Booth et al., 1997). Moreover, those compounds induced the production of reactive oxygen species (ROS) by acting as detergents on membrane enzymes, such as phospholipase A 2, resulting in the formation of prostaglandins and leukotrienes (Bernstein et al., 2005).

Pro-cancerous activity derived from SBA has also been described *in vivo*. In a mouse model, treatment with a carcinogen at the neonatal stage and posterior feeding under a high fat-diet induced the appearance of hepatocellular carcinoma, showing a senescence-associated secretory phenotype (SASP) in hepatic stellate cells (Yoshimoto et al., 2013). The level of DCA produced by enteric bacteria was increased under these conditions, and OTU analysis revealed an increase in DCA-producing bacteria belonging to Firmicutes from *Clostridium* cluster XI (Yoshimoto et al., 2013).

Human studies indicate that African Americans have a higher incidence of and higher mortality from CRC than other ethnic population in the USA (O'Keefe et al., 2007). In a search for possible mechanisms, microbiome compositions between African Americans and native Africans were analyzed; the former group were enriched in *Bacteroides* spp., whereas the later was dominated by *Prevotella* spp. (Ou et al., 2013). This reflected the differences in bacterial enrichment between western and fiber-rich diets. Additionally, genes coding for SBA and fecal SBA concentrations were higher in African Americans, whereas short-chain fatty acids were higher in native Africans (Ou et al., 2013). This scenario suggests that similar genetic backgrounds differ in phenotype and proclivity to develop a certain disease, and this difference is mainly driven by diet and different microbiome conformations.

#### Short chain fatty acids

Consumption of dietary fiber stimulates saccharolytic fermentation by diverse gut microbes that produce short-chain fatty acids (SCFA), mainly acetate, propionate, and butyrate (Holmes et al., 2012). Bacteroidetes produce high levels of acetate and propionate, whereas Firmicutes bacteria produce high amounts of butyrate. Acetate and propionate are found in portal blood and are eventually metabolized by the liver or peripheral tissues (Honda and Littman, 2012). Butyrate is considered a pleiotropic metabolite, functioning as the primary energy source for colonocytes, reducing oxidative stress and inhibiting inflammation (Hamer et al., 2008).

Some anticancer activities have been attributed to butyrate. By functioning as an inhibitor of histone deacetylase (HDAC), butyrate induces hyperacetylation of core histone proteins (H3 and H4) when compared with other SCFA. Among its effects as an HDAC inhibitor, butyrate can induce *in vitro* S-phase arrest of colorectal adenocarcinoma cells and inhibit its growth by inducing apoptosis and the expression of the cell cycle regulators p21 and cyclin B1 (Hinnebusch et al., 2002).

Interestingly, those effects depend on cell status, i.e., normal vs. cancer. In the former, butyrate stimulates proliferation (functioning as an energy source); while in cancerous cells, butyrate inhibits proliferation and induce apoptosis (Comalada et al., 2006). Donohoe et al. analyzed these context-dependent effects from the perspective of the Warburg effect (Donohoe et al., 2012). Due to the Warburg effect, cancer cells primarily depend on aerobic glycolysis instead of oxidative metabolism for survival. In this context, butyrate is not used as an energy source and its accumulation is allowed inside the nuclei, inhibiting HDAC in cancer cells. Experimental inhibition of the Warburg effect in cancerous colonocytes induced cell proliferation, suggesting that the Warburg effect is necessary for observing the butyrate antiproliferative effect (Donohoe et al., 2012).

On the other hand, CRC-prone mice revealed a paradoxical effect of butyrate on colonic cancer cells. By using a mouse model with mutations in the adenomatous polyposis coli (APC) and DNA mismatch repair (MMR) genes (as commonly observed in humans), Belcheva et al. observed an anomalous proliferation of colonic epithelial cells and formation of polyps (Belcheva et al., 2014). Furthermore, using antibiotics or lowering carbohydrates in diet reduced the development of tumors. This indicates an involvement of microbial metabolism and diet in cancer development under this particular host's genetic background. The authors identified butyrate as a causative of disease onset, and the sole administration of butyrate was sufficient to increase polyp number and epithelial cell proliferation. Given the apparently paradoxical effects of butyrate on cancerous phenotypes, there is a potential therapeutic modification of bacterial activities with antibiotics and/or diet modifications for cancer patients in order to improve the outcome.

#### Proteins and red meat diet-associated compounds

When carbohydrates get depleted from the proximal colon, protein fermentation can occur in the distal colon (Windey et al., 2012). This activity is mainly driven by colonic bacteria and results in the production of noxious metabolites such as ammonia, amines, phenols and sulfides. Western diets, provide metabolites like fats, heme and heterocyclic amines, and those are suggested to play a role in CRC development (Windey et al., 2012).

Amino acids fermented by colonic bacteria include lysine, arginine, glycine, and the branched chain amino acids (BCAA) leucine, valine, and isoleucine. This generates a diversity of end products including ammonia, SCFA, and branched-chain fatty acids (BCFA) valerate, isobutyrate, and isovalerate. Microbial metabolism of amino acids can also produce biogenic amines by decarboxylation of amino acids (Windey et al., 2012; Neis et al., 2015).

Bacterial metabolism of aromatic amino acids results in the production of phenolic and indolic compounds that are excreted as p-cresol. *In vitro* studies in epithelial colonic cells have shown detrimental effects and genomic DNA damage by ammonia, sulfides, p-cresol and phenolic compounds (Pedersen et al., 2002; Attene-Ramos et al., 2010; Windey et al., 2012). Hydrogen sulfide also inhibits cellular respiration, at least in part by acting as an inhibitor of cytochrome c oxidase, which participates in the final step to produce ATP. These noxious effects have been associated with Inflammatory bowel disease and cancer (Medani et al., 2011).

Additionally, epidemiological and experimental studies have shown that red meat induces more genetic damage than white meat (Toden et al., 2007). By studying the characteristic compound of red meat, heme molecules, Ijssennagger et al. reported that the colon microbiota facilitates, heme-induced epithelial injury and hyperproliferation as a result of the activity of hydrogen sulfide-producing and mucin-degrading bacteria. They observed that the microbiota facilitates heme-induced hyperproliferation by opening the mucus barrier. Bacterial hydrogen sulfide can reduce the S-S bonds in polymeric mucin, thereby increasing the mucus layer permeability for mucin-degrading bacteria and cytotoxic micelles (Ijssennagger et al., 2015). Antibiotic treatment prevented the heme-induced cell damage and diminished the expression of cell cycle genes.

It has been shown that a small set of metabolites can modify host physiology; however, numerous metabolites in humans have not been investigated (da Silva et al., 2015). Therefore, further research to categorize new metabolites; transport mechanisms and characterize the biotransformation processes by the microbiome, is a top priority to identify biomarkers such as compounds, specific taxonomic components or metagenomic-enriched functions. Integrating these studies with epidemiological, clinical or nutritional data can provide clues for the search for these biomarkers.

### Microbiota-mediated inflammation in cancer

The symbiotic nature of the intestinal host-microbial relationship poses health challenges. The immune system has developed adaptations to contain the microbiome while preserving the symbiotic relationship (Hooper et al., 2012). However, opportunistic invasion of host tissue by resident bacteria has serious health consequences including inflammation. Chronic inflammation and inflammatory factors, such as reactive oxygen and nitrogen species, cytokines, and chemokines, can contribute to tumor growth and spread (Garrett, 2015).

Increasing evidence indicates that colonizing microbes can drive cancer development and progression by direct or indirect effects on host tissues (Gagliani et al., 2014). Pattern recognition receptors (PRR) recognize specific conserved microbial patterns (bacterial cell walls, nucleic acids, motility apparatuses). The most studied PRR related to CRC belongs to the group of intracellular Nod-like receptors (NLR) and Toll-like receptors (TLR). Following microbial sensing, these PRR engage a complex set of signaling proteins that shape the host immune and inflammatory response (Jobin, 2013). Some NLR family members, such as NOD-2, NLRP3, NLRP6, and NLRP12 may play a role in mediating CRC (Garrett, 2015). Mice deficient in NOD-2 showed a proinflammatory microenvironment that enhanced epithelial dysplasia following chemically induced injury (Couturier-Maillard et al., 2013), and those deficient in NLRP6 showed enhanced inflammation-induced CRC formation (Hu et al., 2013).

Activation of TLR results in feed forward loops of activation of NF-κB. Microbes associated with cancer appear to activate NF-κB signaling within the tumor microenvironment. NF-κB was more activated (increased nuclear translocation of the p65 NF-κB subunit) in tumors with a high *Fusobacterium nucleatum* (*F. nucleatum*) abundance in human colorectal cancer (Kostic et al., 2013). NF-κB is a master regulator of the inflammatory response, and it acts in a cell type-specific manner, activating survival genes within cancer cells and inflammation-promoting genes in components of the tumor microenvironment. NF-κB activation is prevalent in carcinomas and is mainly driven by inflammatory cytokines within the tumor microenvironment (Didonato et al., 2012). The FadA adhesin of *F. nucleatum* has also been shown to bind to E-cadherin, activate β-catenin signaling and differentially regulate the inflammatory and oncogenic responses in the colon tissue from patients with adenomas and adenocarcinomas (Rubinstein et al., 2013). *In vitro* studies have also revealed that the Fap2 protein from *F. nucleatum* can help tumor cells evade the immune system by binding the inhibitory receptor TIGIT in natural killer cells and inhibiting their cytotoxic activities (Gur et al., 2015). These observations of tumor zones enriched in *Fusobacterium* indicate that the local microbiome conformation is not random and can play an important role in the pro-cancerous phenotype.

The immune system within the tumor microenvironment is not restricted to the innate cells, which present infectious agents to cells of the adaptive immune system for responding selectively and specifically to them. Some adaptive immune responses can be protumorigenic; for instance, upon contact with specific bacteria, CD4^+^T cells can produce cytokines that promote tumor progression (Gagliani et al., 2014). IL-23, is a cytokine mainly produced by tumor-associated myeloid cells activated by microbial products such as flagellin, promotes tumor growth and progression and development of a tumoral IL-17 response (Grivennikov et al., 2012). Enterotoxigenic *Bacteroides fragilis*, which secretes *B. fragilis* toxin, causes inflammation in humans and triggers colitis and strongly induces colonic tumors in multiple intestinal neoplasia (Min) mice. The enterotoxigenic *B. fragilis* induces STAT3 signaling characterized by a selective T_H_17 response for colonic hyperplasia and tumor formation (Wu et al., 2009). T_H_17 cells produce other cytokines besides IL-17, such as IL-22, another cytokine linked to human colon cancer by activation of STAT3 (Jiang et al., 2013).

Notably, inflammation can be associated with other malignant phenotypes that can synergistically act as risk factors for cancer development. For instance, obesity can also generate overrepresentation of bacterial species that produce pro-carcinogenic metabolites, such as SBAs (Louis et al., 2014). Dysbiosis present in obese individuals alters the gut epithelial barrier, making it more permeable to microbial products that activate immune cells in the lamina propria; and reach the liver via the portal circulation, this contributes to the production of proinflammatory cytokines, such as TNF and IL-6 (Font-Burgada et al., 2016). Barrier deterioration was shown to be a major contributor to colorectal tumorigenesis by microbial products that trigger tumor-elicited inflammation (Grivennikov et al., 2012).

## Integrative analysis and the challenges in systems biology

Given that cancer can be produced by a myriad of genetic and environmental factors, understanding its mechanisms and designing optimal treatments calls for computational schemes capable of integrating heterogeneous HT data to move toward personalized and predictive medicine. Among these factors, the microbiome composition in patients constitutes an important component to induce carcinogenesis or other dysfunctional states in human tissues (Thomas and Jobin, 2015).

An explanation of how the microbiome contributes to the physiological state in the host emerged by noticing that microbes are metabolic partners, for which the nutritional habits of the host can induce the dysregulation of biological processes and consequently alter the phenotypic state. For instance, foods enriched in phosphatidylcholine, choline or carnitine, such as red meat and fatty foods, can be metabolized by gut microbes to produce trimethylamine. The liver enzymes can further produce trimethylamine-N-oxide (TMAO), and this metabolite has proatherogenic properties (Koeth et al., 2013). Knowledge about the microbiome composition and levels of its derived metabolite TMAO predicted the probability of suffering a cardiovascular problem, by means of platelet hyperresponsiveness. Even more, the thrombosis potential was transmissible as a microbiome-dependent trait (Zhu et al., 2016). In the case of type 2 diabetes, fasting plasma concentrations of branched chain (BCAA) and aromatic amino acids were higher in people who developed diabetes, and this signature was predictive of developing the disease for more than a decade later (Wang et al., 2011). Interestingly, a metagenomic signature identified in fecal samples from patients with diabetes was the enrichment in metabolic pathways for transport of BCAA and oxidative stress (Qin et al., 2012). It to expect in the near future, that identification of cancer biomarkers, microbiome signatures and its implementation in mechanistic models will also aid in predicting cancer risk and prognosis.

Thus, microbiota metabolism is a cornerstone for maintaining human and microbial symbiosis, whose involvement in signaling transduction and transcriptional regulation is capable of inducing wellness or disease in the human body (Chubukov et al., 2014). More importantly, the heterogeneous composition observed in individual microbiota provides evidence for the usefulness of personalized studies in terms of genetic backgrounds, lifestyle, nutrition and environmental factors. Even though these findings are currently supported with experimental evidence, the understanding of how a community of organisms consume and interchange their metabolic and cross-signaling products and how this dynamical behavior influences the phenotypic state of the human host is still an open question.

To decode this bewildering complexity and uncover their underlying mechanisms, combined strategies with available data coming from different HT technologies and conceptual schemes from systems biology have been employed. Currently, in systems biology, some paradigms have been suggested to reach this combined description, including genome scale metabolic reconstructions and constraints-based modeling (Bordbar et al., 2014). The implementation of this paradigm has made it possible to explore the metabolic phenotypes of isolated microorganisms and has successfully contributed to areas such as *in vitro* microbial evolution and organisms with biotechnological and therapeutic applications (Resendis-Antonio et al., 2007; Bordbar et al., 2014). More fundamentally, these schemes have served as a guide to characterize the metabolic activity of human tissues and explore the metabolic phenotypes in cancer (Resendis-Antonio et al., 2010; Lewis et al., 2012). Remarkably, genome scale metabolic reconstruction and computational modeling have extended the scope. Currently, it is possible to model the metabolic interaction between different tissues in the human body (Bordbar et al., 2011), and new approaches are currently pointing toward the integration of models for human-microbiome interaction to explore the metabolic activity in a community of microorganisms (Heinken and Thiele, 2015; Shoaie et al., 2015). Notably, these approaches pave the path toward quantitative models able to predict the metabolic profile in a community of microorganisms and exploring the mechanisms by which their metabolic products could drive the development of cancer.

Although this is a titanic enterprise, systems biology is a cornerstone in precision medicine for moving toward: (1) the coherent interpretation of heterogeneous HT data; (2) identification of potential biomarkers in cancer; and (3) the optimal design of personalized treatments in clinical trials (Wang R.-S. et al., 2015). Among the immediate challenges needing to be overcome to materialize those aims, the development of integrative conceptual schemes of HT data is important. Nonetheless, its capacity to provide meaningful biological insight will be the proof of concept. The development of methods for a coherent interpretation of data is particularly important in cancer studies where massive genome characterization of a variety of cancers have been reported (Cancer Genome Atlas Network, 2015). The accumulation of enormous quantities of molecular data has led to the emergence of systems biology as a set of principles that underlie the base functional properties of living organisms, evaluating and interpreting interactions between molecules (Kristensen et al., 2014). From a systems biology perspective, the use of genomic technologies and computational procedures may provide molecular approaches to early disease detection and opportunities for identifying high-risk individuals, thus contributing to opportune diagnosis (Stewart et al., 2015). In terms of cancer and the microbiome, the computational platform from systems biology should be able to integrate HT data such as metagenome and metatranscriptome data for building hypotheses of host-microbiota metabolic activity, and eventually evaluate its role in cancer development (Bäckhed et al., 2012). The hypothesis generated using this approach can be contextualized with the nutritional information of patients, genetic variability, immune status or clinical record. As stated before, integrating microbiome analysis and host data has the potential to predict the disease outcome and has recently been explored in microbiome-related diseases.

Studying the microbiome variation over time offers an exceptional window to understand the properties leading to health and disease states. To date few longitudinal microbiome studies have been conducted on humans, mainly using 16S rRNA sequencing and observing changes in microbial diversity for over a year (Caporaso et al., 2011; David et al., 2014). Results from whole shotgun metagenomics over time are consistent with 16S studies, indicating both small taxonomic and functional variation over time in the absence of perturbations (Voigt et al., 2015). Although those whole shotgun metagenomic studies are scarce, it is expected that price reduction on sequencing will promote their application. From early exposition at birth to adulthood, factors such as diet, immunological tolerance, environment and microbe-microbe interactions can account preferred taxonomic compositions (Wu et al., 2011; Costello et al., 2012; Nutsch et al., 2016). Despite these factors can include an stochastic component, robustness is observed in tissue-specific microbiome identities maintained over time (Caporaso et al., 2011). Notably, when the community suffers a perturbation, taxonomically related bacteria are preferred as substitutes and subject-specific proportions are maintained within the same taxa (David et al., 2014).

Understanding the principles that rule the microbiome dynamics is an important challenge for system biology, nonetheless new paradigms capable to integrate data bases (Hood et al., 2014; Integrative HMP Research Network Consortium, 2014), empirically dissected patterns (Caporaso et al., 2011; David et al., 2014), and computational models (Stein et al., 2013; Mcgeachie et al., 2016) can aid to reach this enterprise. An hypothesis to explore in future is the idea of early warning signals that could link the dynamical microbiome behavior preceding the progression of a human disease (Faust et al., 2015). The advance in this aim will have a strong impact to translate basic knowledge into precision medicine.

In summary, systems biology suggests that human diseases are fundamentally a system issue at which our phenotype (functional or dysfunctional) is an emergent property that results from host-microbiome interactions. Understanding how this property emerges at a molecular level is valuable to reach one of the aims in precision medicine: the desire for more effective treatments in cancer based on personalized genetic background and lifestyle. In this context, HT technologies and biochemical, physiological and clinical data can be organized and evaluated using a network approach that can be useful for predicting disease expression or response to therapies (Loscalzo and Barabasi, 2011). Finally, addressing these aims will contribute positively to understanding the biological mechanisms in human diseases, and providing the right treatment for the right patients at the right moment with clinical strategies based on genomic, proteomics, metabolomics, and taking into account the behavioral and environment background information of individual patients. All these schemes aim to improve diagnostic power.

## Toward the clinical applications of host-microbiome interactions in cancer

The development of diagnostic tests using biomarkers to be applied for early detection is likely a key aspect for precision medicine. For example, the immunosignature approach leverages the response of antibodies to disease-related changes and can be used for the simultaneous classification of multiple cancers (Stafford et al., 2014). In addition, researchers have evaluated the potential of the fecal microbiota for early-stage detection of CRC and as a screening tool to differentiate between healthy, adenoma, and carcinoma clinical groups (Zackular et al., 2014). Using metagenomic sequencing, it is possible to identify microbiome signatures able to distinguish CRC patients from tumor-free controls (Zeller et al., 2014).

Conversely, germ-free status and treatment with antibiotics has been shown to lead to a reduction of the numbers of tumors in genetic experimental models of CRC, suggesting the use of antibiotics to knock out cancer-promoting gut microbes (Schwabe and Jobin, 2013; Thomas and Jobin, 2015). For instance, cefoxitin treatment resulted in complete clearance of enterotoxigenic *Bacteroides fragilis*, a microbe that causes IL17A-dependent colon tumors. *Bacteroides fragilis* eradication reduced tumorigenesis and decreased mucosal IL-17A expression (DeStefano Shields et al., 2016). Nonetheless, clinical studies must be developed to probe the clinical effectiveness and the potential effect on the whole human microbiome.

Other players must be taken into account in shaping the microbiome. From environmental studies, it has been established that bacteriophages shape bacterial community structure and function via predation and gene transfer (Chibani-Chennoufi et al., 2004). In contrast to antibiotics, lytic phages are fairly specific, usually only targeting a subgroup of strains within one bacterial species, for treating bacterial human diseases. For instance, when a bacteriophage cocktail was used to treat *Shigella sonnei* in a mouse model, bacteriophage administration significantly reduced *Shigella* colonization without deleterious side effects and distortions in the gut microbiota (Mai et al., 2015). Taking this into account, using bacteriophages has been proposed to target specific strains of bacteria that are implicated in cancer, while leaving the rest of the microbiome unchanged (DeWeerdt, 2015).

In addition, with diet being a key determinant shaping the gut microbiome, dietary interventions and probiotics that promote the development of microorganisms providing health benefits are an attractive way to prevent or treat diseases such as cancer. Dietary interventions, such as a curcumin-supplemented diet increased survival and entirely eliminated tumor burden in a mouse model of colitis-associated colorectal cancer. The beneficial effect of curcumin on tumorigenesis was associated with the maintenance of a more diverse colonic microbial ecology (Mcfadden et al., 2015). Furthermore, dietary intervention with polyphenol extracts modulate the human gut microbiota toward a more healthy profile increasing the relative abundance of bifidobacteria and lactobacilli (Marchesi et al., 2015). The beneficial effects of natural polyphenols and their synthetic derivatives are extensively studied in context of cancer prophylaxis and therapy (Lewandowska et al., 2016).

**Figure 4:**
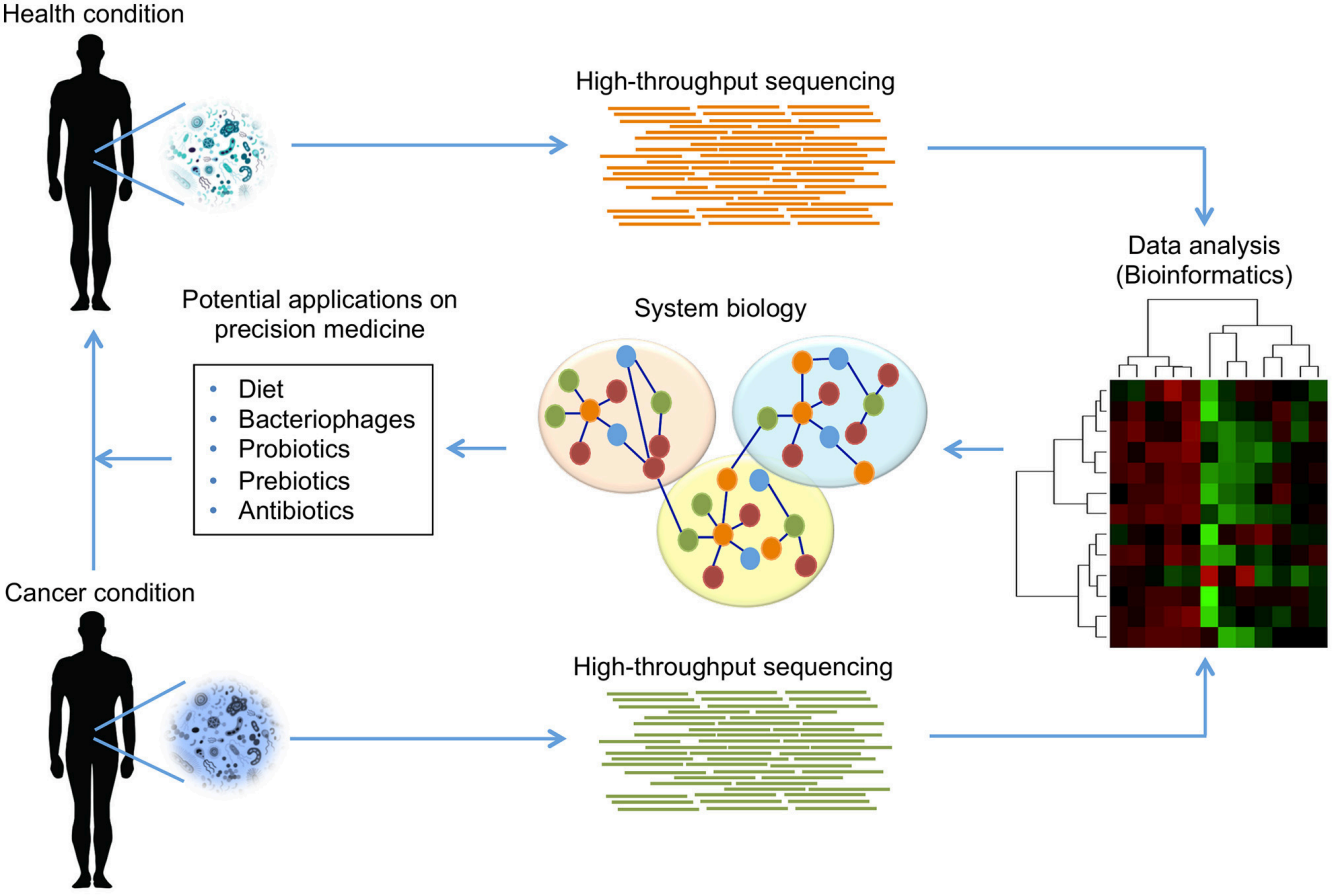
**Modulation of human microbiome composition as potential treatment in cancer**. The use of HT sequencing technologies can provide detailed information about the taxonomic composition and the functional capabilities of microbial communities found in humans. Using these technologies, it is possible to identify those communities that are present or absent in a health condition comparing with cancer condition. The access to microbiome data, and its analysis by bioinformatics tools, allows establishing integrative models using a systems biology approach, which offers an opportunity to propose potential strategies for treatment in cancer. The evidence suggests that diet, bacteriophages, probiotics, prebiotics and antibiotics can modulate human microbiome to reduce microbial dysbiosis, eliminate pathogenicity in cancer condition, and promote beneficial effects leading a health condition.

In terms of reducing gastrointestinal inflammation and preventing CRC, beneficial roles of probiotics have been demonstrated. Moreover, a novel probiotic mixture suppressed hepatocellular carcinoma growth in mice; shotgun-metagenome sequencing revealed the crosstalk between gut microbial metabolites and hepatocellular carcinoma development (Li et al., 2016). Probiotics shifted the gut microbial community toward certain beneficial bacteria, including the genera *Prevotella* and *Oscillibacter*, which are producers of anti-inflammatory metabolites (Li et al., 2016; Figure 4).

Another area of clinical implications of the microbiome relates to its influence on the host's immune system response against pathogens and cancer (Abt et al., 2012; Belkaid and Hand, 2014). For instance, using antibiotics, the reduction of intestinal microbes ablated the effect not only of the immunotherapy directed to TLRs but also the effectiveness of platinum chemotherapy (Iida et al., 2013). Another type of immunotherapy against cancer relies on immune-checkpoint blockers (ICB). Both Cytotoxic T-Lymphocyte Antigen-4 (CTLA-4) and Programmed Death-1 (PD-1) are receptors that dampen T cell responses, and blocking these receptors with antibodies is approved for patients with advanced melanoma to enhance its recognition and elimination (Rotte et al., 2015). *In vivo* studies have shown that CTLA-4 blockade reduces tumor growth in specific pathogen-free mice but not in germ-free mice. This effect relied on the presence of the gut intestinal microbiota and the activation of both CD4+ T_H_1 cells and dendritic cells (DCs). Moreover, in melanoma patients who responded to anti-CTLA-4 treatment, the abundance in the stool of *Bacteroides thetaiotaomicron* and *B. fragilis* correlated with the response to therapy. This protective effect can be transferred to mice by a fecal microbial transplant (FMT) (Vétizou et al., 2015). In addition, Sivan et al. found that differences in the gut intestinal microbiome composition in mice of the same strain can alter the response to PD-1 blockade, with stool samples enriched in *Bifidobacterium* spp. having a robust CD8+ T cell tumor infiltration and DC activation. The protective effect was also transferred between mice by means of FMT (Sivan et al., 2015).

Those results indicate an important modulatory role of both the microbiota activities and composition on the immune response to cancer. Importantly, the effectiveness of treatment in those experiments depended on the integrity of the gut intestinal microbiome, but its effects extend to the systemic level. From a translational point of view, the manipulation of the microbiome composition by means of probiotics, prebiotics or even FMT can have therapeutic benefits in cancer treatment.

## Information management in precision medicine

Analysis of massive amounts of data generated by HT technology and personal clinical records requires computational capacities to handle this data, and, as a consequence, unveil the biological information of interest. Data collection, storage, and handling, and privacy policies of personalized genome data becomes a central issue that must be solved. Information management faces different challenges that can be classified into three aspects: storage, structural organization, and safety. The storage problems have been solved by the buying or leasing of space in the cloud hosting systems of large technology companies that simultaneously have been developing applications for data analysis. The structural organization involves the appropriate classification of personal records and HT data and the development of an efficient and optimized mechanism to look for the desired information through heterogeneous sources of biological databases.

Finally, given the social, ethical and legal implications of personalized information, it should be stored in a protected way. To this end, the management system can include protocols for prevention and protection, access control and a plan of action to prevent the loss of information when some event endangers the integrity and security of the data (https://www.whitehouse.gov/sites/whitehouse.gov/files/documents/PMI_Security_Principles_and_Framework_FINAL_022516.pdf).

## Perspectives

Compositional and functional alterations of the human microbiome have been related to the development of complex diseases such as cancer, type 2 diabetes and obesity. As previously mentioned, host-microbiome interactions play a major role in determining the metabolic phenotype in the host, and, more importantly, their particular composition can serve as a potential measurement for establishing wellness and monitoring the evolution of diseases. This notion has not only changed our paradigm of how our body works, like a superorganism, but also unveiled the outstanding role that microorganisms play in establishing wellness or disease states. Although outstanding breakthroughs have been accomplished to discover its connection, new conceptual schemes able to integrate innovative HT technologies and computational modeling are required to improve our measurements and elucidate their fundamental mechanisms.

For instance, single-cell genomics has the potential to assemble the genomes of viruses and microorganisms that are at low frequencies, thus contributing to a better characterization of the biological samples (Gawad et al., 2016). There has been extraordinary progress in single-cell DNA and RNA sequencing for cancer research, specifically regarding evolution, diversity of cells in tumor progression, and intra-tumor heterogeneity depending on spatial localization of single cancer cells in tissue sections (Crosetto et al., 2015; Navin, 2015; Gawad et al., 2016). In the context of host-microbiome interactions, using the spatial information of the surrounding microbiome state and measurements of intra-tumor genetic heterogeneity might have prognostic utility for predicting which patients will be more likely to show poor response to therapy, higher probability of metastasis, or poor overall survival (Burrell et al., 2013; Murugaesu et al., 2013; Almendro et al., 2014). These and other HT technologies permit us to characterize the microbiome composition and open the possibility of therapeutic applications with a focus on precision medicine: the notion of a precision medicine with treatments applied at the right time, at the right dose, and for the right patient.

Precision medicine based on powerful HT technologies for characterizing patients, such as genomics, proteomics and metabolomics, and computational tools for analyzing large sets of data will integrate the discovery of biomarkers and the electronic medical records to provide evidence for the improvement of clinical practice. The big challenge of data analysis of HT technologies is the development of new computational algorithms to improve the integration of the information from different platforms. In this context, deep learning and machine learning have been proposed as good alternatives to perform these tasks (Eddy, 2009; https://arxiv.org/pdf/1603.06430.pdf). In addition, ambitious projects in precision medicine need to leverage important resources, such as research cohort biobanks for longitudinal research studies, and an efficient bioinformatics system that aids in the translation from biomedical research to molecular targeting and identification of biomarkers that correlate with the disease state. Intensive investigations are being conducted to illustrate how microbiome profiles, taking into account relationships with the host, could be used as biomarkers to revolutionize prognostication in cancer.

However, the interindividual variations in microbiome composition can potentially influence cancer evolution and the effectiveness of treatment. Cross sectional studies in large cohorts showed no evidence of a “core” of OTUs shared among healthy subjects (Huse et al., 2012). This highlights geography, ancestry, diet and age as crucial factors shaping the microbiome composition (Yatsunenko et al., 2012). On the other hand, at the metagenomic level, several functions or pathways are more consistent across individuals than taxonomic composition (HMP Consortium, 2012b; Knights et al., 2014). These evidence suggests both a robustness in functions and membership redundancy in the microbiome.

Harnessing the microbiome to improve cancer diagnosis and treatment is challenging given this interindividual variation. As we are still uncovering the mechanisms behind the emergent phenotypes from host-microbiome interactions, profiling the microbiome composition and its functional properties (i.e., metatranscriptomics and metabolomics) can provide more insight on the significance of different compositions in different states (Shade and Handelsman, 2012; Shafquat et al., 2014). Nonetheless, identifying conserved or consistently altered functions of the microbiome can also be elusive. A comparison of functional alterations at the metagenomic level revealed some overlapping, but no universal biomarkers between cohorts with type 2 diabetes (Qin et al., 2012; Karlsson et al., 2013). Thus, characterizing microbiome biomarkers should take into account the specific traits of the populations under study.

Finally, precision medicine faces many other challenges that will be addressed not only from a scientific point of view but also from a social and ethical point of view, including the proper distribution of the benefits of these technologies across most regions of the world and the development of reliable computational platforms that allow data to be stored confidentially, private and protected. Likewise, coordination with ethics committees and regulation will also be necessary for the use of information without the risk of infringement of the patient's rights and to understand and regulate the legal, social, and economic implications.

## Author contributions

All authors contributed extensively to the design and discussions of the material presented in this review. All the authors participated to wrote the final version of the paper.

## Funding

The authors thank the financial support from the Research Chair on Systems Biology-FUNTEL and Instituto Nacional de Medicina Genomica, Mexico.

### Conflict of interest statement

The authors declare that the research was conducted in the absence of any commercial or financial relationships that could be construed as a potential conflict of interest. The reviewer AA and handling Editor declared their shared affiliation, and the handling Editor states that the process nevertheless met the standards of a fair and objective review.
