# Supplementary material for: Host-Microbiome Interaction and Cancer: Potential Application in Precision Medicine
Source: Front Physiol. 2016 Dec 9;7:606. doi: 10.3389/fphys.2016.00606 (PMC5145879; doi:10.3389/fphys.2016.00606)
Supplement: Supplementary file 1 [file Table1.DOCX]

**Host-microbiome interaction and cancer: potential application in precision medicine.**

**Alejandra V. Contreras^3,4^, Benjamin Cocom-Chan^1,3,4^, Georgina Hernandez-Montes^2^, Tobias Portilllo-Bobadilla^2^ and Osbaldo Resendis-Antonio^1,2,3 *^**

^1^ Human Systems Biology Laboratory & ^2^ Coordinación de la Investigación Científica, Red de Apoyo a la Investigación-UNAM.

^3^ Instituto Nacional de Medicina Genómica.

^4^ These authors contributed equally to this work.

*** Correspondence:** resendis@ccg.unam.mx / [oresendis@inmegen.gob.mx](mailto:oresendis@inmegen.gob.mx)

# Supplementary Figures and Tables

**Supplementary Table 1**. Bioinformatics software for metabolomics and microbiome studies.

| Software or package | Description | Reference |
| --- | --- | --- |
| ABySS | A parallelized sequence assembler for short read sequence data. | [(Simpson et al., 2009)](https://paperpile.com/c/LXYLtZ/KvZo) |
| bcl2fastq | Conversion Software owen by illumina. | Illumina, Inc |
| bioOTU | An improved method (bioOTU) that first assigns taxonomy to unique tags at genus level for separating the error-free sequences of known species in reference database from artifacts, and then cluster them into OTUs. | [(Chen et al., 2016)](https://paperpile.com/c/LXYLtZ/RUu0) |
| BOTUX | A tool for the classification of 16S rRNA gene sequences into OTUs that uses bayesian probabilistics models. | [(Koparde et al., 2014)](https://paperpile.com/c/LXYLtZ/IJKS) |
| CD-Hit | A protein sequence clustering program. | [(Fu et al., 2012; Li and Godzik, 2006)](https://paperpile.com/c/LXYLtZ/rYDP+bvO2) |
| CLARK | A method for classification of metagenomic and genomic sequences using discriminative k-mers. | [(Ounit et al., 2015)](https://paperpile.com/c/LXYLtZ/feaW) |
| CLUSTOM | A novel method for clustering 16S rRNA next generation sequences by overlap minimization. | [(Hwang et al., 2013)](https://paperpile.com/c/LXYLtZ/PQaw) |
| COMAN | A web server for comprehensive metatranscriptomics analysis, from quality control of reads to network analysis and visualization. | [(Ni et al., 2016)](https://paperpile.com/c/LXYLtZ/tr3i) |
| DNACLUST | A clustering tool specifically designed for clustering highly-similar DNA sequences. | [(Ghodsi et al., 2011)](https://paperpile.com/c/LXYLtZ/V6yw) |
| ESPIRIT-Tree | A computational algorithm that allows researchers to perform taxonomy analysis, removes low quality reads, groups reads into OTUs and estimates species richness. | [(Cai and Sun, 2011)](https://paperpile.com/c/LXYLtZ/WUX2T) |
| FastQC | A quality control tool for high throughput sequence data. | Babraham Institute |
| FASTX-Toolkit | FASTQ/A short-reads pre-processing tools. | Hannon Lab at Cold Spring Harbor Laboratory |
| flagme | Analysis of Metabolomics GC/MS Data. | Robinson M and Romoli R (2015). flagme: Analysis of Metabolomics GC/MS Data. R package version 1.26.0. |
| FragGeneScan | A gene prediction method FragGeneScan, which combines sequencing error models and codon usages in a hidden Markov model to improve the prediction of protein-coding region in short reads. | [(Rho et al., 2010)](https://paperpile.com/c/LXYLtZ/9vF0W) |
| GhostKOALA | Automatic annotation servers for genome and metagenome sequences, which perform KO (KEGG Orthology) assignments to characterize individual gene functions and reconstruct KEGG pathways, BRITE hierarchies and KEGG modules to infer high-level functions of the organism or the ecosystem. | [(Kanehisa et al., 2016)](https://paperpile.com/c/LXYLtZ/4SBJb) |
| Glimmer-MG | A metagenomics gene prediction system. For finding the protein-coding genes within the sequences. | [(Kelley et al., 2012)](https://paperpile.com/c/LXYLtZ/CyffP) |
| Greengenes database | A 16S rRNA gene database. | [(DeSantis et al., 2006)](https://paperpile.com/c/LXYLtZ/AVnf) |
| HMMER | A web server for searching sequence homologs in databases, and for making sequence alignments using probabilistic profile hidden Markov models. | [(Finn et al., 2011)](https://paperpile.com/c/LXYLtZ/WsXx) |
| Human Metabolome Database | Database containing detailed information about small molecule metabolites found in the human body. The database contain three kinds of data: 1) chemical data, 2) clinical data, and 3) molecular biology/biochemistry data. | [(Wishart et al., 2012)](https://paperpile.com/c/LXYLtZ/eBNQ) |
| IDBA-UD | An algorithm that is based on the de Bruijn graph approach for assembling reads from single cell sequencing or metagenomic sequencing technologies with uneven sequencing depths. | [(Peng et al., 2012)](https://paperpile.com/c/LXYLtZ/FNBV) |
| IDBA-MT | Assembler for meta-transcriptome based on De Bruijn Graphs. | [(Leung et al., 2013)](https://paperpile.com/c/LXYLtZ/vBRe) |
| IMG/M | A systems provide support for annotation and expert review of unpublished metagenomic data sets. | [(Markowitz et al., 2006, 2014)](https://paperpile.com/c/LXYLtZ/OZGvW+qUgYg) |
| iMOP | Initiative on model organism proteomes. | [(Heazlewood et al., 2015)](https://paperpile.com/c/LXYLtZ/NDEsi) |
| JCVI METAREP | A Web 2.0 application designed to help scientists analyze and compare annotated metagenomics datasets, provides graphical summaries for top taxonomic and functional classifications as well as a GO, NCBI Taxonomy and KEGG Pathway Browser. | [(Goll et al., 2010)](https://paperpile.com/c/LXYLtZ/uY3Lj) |
| KEGG | Kyoto Encyclopedia of Genes and Genomes is a knowledge base for systematic analysis of gene functions, linking genomic information with higher order functional information. | [(Ogata et al., 1999)](https://paperpile.com/c/LXYLtZ/06qpe) |
| MAIT | Statistical Analysis of Metabolomic Data. | [(Fernandez-Albert et al., 2014)](https://paperpile.com/c/LXYLtZ/LdvEZ) |
| MaSuRCA | A whole genome assembly software that combines the efficiency of the de Bruijn graph and Overlap-Layout-Consensus (OLC) approaches. | [(Zimin et al., 2013)](https://paperpile.com/c/LXYLtZ/peQb) |
| MEGAHIT | A de novo assembler for assembling large and complex metagenomics data in a time- and cost-efficient manner. | [(Li et al., 2015, 2016)](https://paperpile.com/c/LXYLtZ/Smihm+JSYae) |
| MEGAN | A computer program that allows laptop analysis of large metagenomic data sets, to compute and explore the taxonomical content of the data set; to interactively analyze and compare metagenomic and metatranscriptomic data. | [(Huson and Weber, 2013)](https://paperpile.com/c/LXYLtZ/JDCV) |
| Metab | An R Package for analyses of GC-MS data. | [(Aggio et al., 2011)](https://paperpile.com/c/LXYLtZ/yUkVH) |
| MetaboAnalyst | Tool for metabolomic data processing, normalization, multivariate statistical analysis and data annotation. | [(Xia et al., 2009)](https://paperpile.com/c/LXYLtZ/yCzi) |
| metabomxtr | A package to run mixture models for truncated metabolomics data with normal or lognormal distributions. | [(Nodzenski et al., 2014)](https://paperpile.com/c/LXYLtZ/ug2r1) |
| MetaCyc | A comprehensive and freely accessible resource for metabolic pathways and enzymes from all domains of life. | [(Liu et al., 2013a)](https://paperpile.com/c/LXYLtZ/cQrA) |
| MetaGeneMark | The MetaGeneMark plugin is optimized for gene finding in bacterial genomes and metagenomes. | GENE PROBE Inc. |
| MetaGUN | A gene prediction software for metagenomic fragments based on a machine learning approach of SVM. | [(Liu et al., 2013b)](https://paperpile.com/c/LXYLtZ/2VqQ) |
| MetAMOS | A metagenomic assembly and analysis pipeline; from sequencing reads or assemblies to final charts and HTML reports. | [(Treangen et al., 2013)](https://paperpile.com/c/LXYLtZ/dIlF) |
| metaMS | MS-based metabolomics annotation pipeline. | [(Wehrens et al., 2014)](https://paperpile.com/c/LXYLtZ/956vS) |
| MetaORFA | A pipeline to assembling metagenomic sequencing reads. | [(Ye and Tang, 2009)](https://paperpile.com/c/LXYLtZ/WmgP) |
| MetaPlAn2 | MetaPhlAn is a computational tool for profiling the composition of microbial communities from metagenomic shotgun sequencing data. | [(Segata et al., 2012)](https://paperpile.com/c/LXYLtZ/AHbV) |
| MetaQUAST | MetaQUAST, a modification of QUAST, the state-of-the-art tool for genome assembly evaluation based on alignment of contigs to a reference. | [(Mikheenko et al., 2016)](https://paperpile.com/c/LXYLtZ/KNJwk) |
| MetaTrans | An open-source pipeline for metatranscriptomics; from quality control to taxonomic and functional analysis. | [(Martinez et al., 2016)](https://paperpile.com/c/LXYLtZ/MXQa7) |
| MetaVelvet | A de novo assembler of genomes and metagenomes. The last version MetaVelvet-SL improves the classification of chimeric nodes using supervised machine learning. | [(Afiahayati et al., 2015; Namiki et al., 2012)](https://paperpile.com/c/LXYLtZ/tp0l+Rajw) |
| metaX | An R package for metabolomic data analysis. | Wen B (2015). metaX: An R package for metabolomic data analysis. R package version 1.0.3. |
| METLIN | The Scripps Center for Metabolomics is pleased to announce XCMS Online, a new user-friendly variant of XCMS for processing metabolomics data without installation. | https://metlin.scripps.edu/index.php |
| MG-RAST | A web accessible system that provides a suite of tools for analysis and visualization of metagenomic data. | [(Keegan et al., 2016; Wilke et al., 2016)](https://paperpile.com/c/LXYLtZ/BEJlQ+FQipi) |
| MICCA | MICrobial Community Analysis is a software pipeline for the processing of amplicon sequencing data, from raw sequences to OTU tables, taxonomy classification and phylogenetic tree inference. The pipeline can be applied to a range of highly conserved genes/spacers, such as 16S rRNA gene, Internal Transcribed Spacer (ITS) and 28S rRNA. | [(Albanese et al., 2015)](https://paperpile.com/c/LXYLtZ/evtsa) |
| MIMOSA | A pipeline for joint metabolomics analysis and taxonomic composition from microbial communities. It addresses the contribution of community composition on metabolome profiles to identify key taxa and genes contributing to the metabolite shifts. | [(Noecker et al., 2016)](https://paperpile.com/c/LXYLtZ/Q2IH) |
| MOCAT2 | A software pipeline for metagenomic sequence assembly and gene prediction with novel features for taxonomic and functional abundance profiling. | [(Kultima et al., 2016)](https://paperpile.com/c/LXYLtZ/sTrv) |
| Mothur | A complete software package to analyze community sequence data; to trim, screen, and align sequences, calculate distances, assign sequences to operational taxonomic units and describe the α and β diversity. | [(Schloss et al., 2009)](https://paperpile.com/c/LXYLtZ/L3cF) |
| mOTU | A method for taxonomic composition profiling of environmental samples. | [(Sunagawa et al., 2013)](https://paperpile.com/c/LXYLtZ/7PO2) |
| M-pick | M-pick, a modularity-based method for OTU picking of 16S rRNA sequences. | [(Wang et al., 2013)](https://paperpile.com/c/LXYLtZ/Fdqb) |
| MtHc | MtHc: a motif-based hierarchical method for clustering massive 16S rRNA sequences into OTUs. | [(Wei and Zhang, 2015)](https://paperpile.com/c/LXYLtZ/tQb4) |
| mQTL.NMR | Metabolomic Quantitative Trait Locus Mapping for 1H NMR data. | Hedjazi L and Cazier J (2015). mQTL.NMR: Metabolomic Quantitative Trait Locus Mapping for 1H NMR data. R package version 1.4.0 |
| MUSiCC | A software package for normalizing and correcting gene abundance measurements derived from metagenomic shotgun sequencing. It uses a large set of universal single-copy genes to obtain an accurate abundance of all other genes. | [(Manor and Borenstein, 2015)](https://paperpile.com/c/LXYLtZ/Ymad) |
| MZmine | MZmine 2 is an open-source software for mass-spectrometry data processing, with the main focus on LC-MS data. | [(Katajamaa et al., 2006)](https://paperpile.com/c/LXYLtZ/hgBGx) |
| Omega | An overlap-graph metagenome assembler of microbial communities. | [(Haider et al., 2014)](https://paperpile.com/c/LXYLtZ/0dWR) |
| Orphelia | A gene prediction algorithm for metagenomic fragments based on a two-stage machine learning approach. | [(Hoff et al., 2008)](https://paperpile.com/c/LXYLtZ/p5rO) |
| PAPi | Predict metabolic pathway activity based on metabolomics data. | [(Aggio, 2014)](https://paperpile.com/c/LXYLtZ/DVx2) |
| Phymm and PhymmBL | A classification software designed for metagenomics experiments that assigns taxonomic labels to short DNA reads. | [(Brady and Salzberg, 2009, 2011)](https://paperpile.com/c/LXYLtZ/mUo3+rBsm) |
| Phyloseq | A software project, phyloseq, dedicated to the object-oriented representation and analysis of microbiome census data in R. | [(McMurdie and Holmes, 2014; McMurdie and Susan, 2013)](https://paperpile.com/c/LXYLtZ/gq2zs+mtYe0) |
| PICRUSt | Phylogenetic Investigation of Communities by Reconstruction of Unobserved States (PICRUSt) is a computational approach to predict the functional composition of a metagenome using marker gene data and a database of reference genomes. | [(Langille et al., 2013)](https://paperpile.com/c/LXYLtZ/ZcJsW) |
| poRe | A package for R that enables users to manipulate, organize, summarise and visualize MinION nanopore sequencing data. | [(Watson et al., 2014)](https://paperpile.com/c/LXYLtZ/jyNb2) |
| poretools | A flexible toolkit for exploring datasets generated by nanopore sequencing devices from MinION for the purposes of quality control and downstream analysis. | [(Loman and Quinlan, 2014)](https://paperpile.com/c/LXYLtZ/L1uY3) |
| QIIME | An open-source bioinformatics pipeline for performing microbiome analysis from raw DNA sequencing data. Includes demultiplexing and quality filtering, OTU picking, taxonomic assignment, and phylogenetic reconstruction, and diversity analyses and visualizations. | [(Caporaso et al., 2010; Navas-Molina et al., 2013)](https://paperpile.com/c/LXYLtZ/GARqO+lAUam) |
| RiboFR-Seq | RiboFR-Seq (Ribosomal RNA gene flanking region sequencing), for capturing both ribosomal RNA variable regions and their flanking protein-coding genes simultaneously, can link the annotations of 16S rRNA and metagenomic contigs to make a consensus classification. | [(Zhang et al., 2016)](https://paperpile.com/c/LXYLtZ/4KLfP) |
| SGA | An assembler that uses the overlap-based string graph model designed for mammalian-sized genomes. | [(Simpson and Durbin, 2012)](https://paperpile.com/c/LXYLtZ/lCH6) |
| SILVA database | A comprehensive web resource for up to date, quality-controlled databases of aligned ribosomal RNA (rRNA) gene sequences and online services. | [(Quast et al., 2013)](https://paperpile.com/c/LXYLtZ/Etemh) |
| ShotgunFunctionalizedR | An R-package for functional comparison of metagenomes. The package contains tools for importing, annotating and visualising metagenomic data produced by shotgun high-throughput sequencing. | [(Kristiansson et al., 2009)](https://paperpile.com/c/LXYLtZ/2e9TW) |
| Sickle | A windowed adaptive trimming tool for FASTQ files using quality. | https://github.com/najoshi/sickle |
| SMART | A metagenomics classification algorithm for microorganisms as well as large plant, mammalian, or invertebrate genomes. | [(Lee et al., 2016)](https://paperpile.com/c/LXYLtZ/8uCb) |
| SortMeRNA | A software designed to rapidly filter rRNA fragments from metatranscriptomic data. | [(Kopylova et al., 2012)](https://paperpile.com/c/LXYLtZ/7Dfg) |
| SPAdes | An assembler for both single-cell and standard (multicell) assembly that is an excellent option for bacterial genomes. | [(Bankevich et al., 2012)](https://paperpile.com/c/LXYLtZ/Mlin) |
| specmine | Provides a set of methods for metabolomics data analysis, including data loading in different formats, pre-processing, metabolite identification, univariate and multivariate data analysis. | [(Costa et al., 2016)](https://paperpile.com/c/LXYLtZ/9hsX) |
| STAMP | A graphical software package that provides statistical hypothesis tests and exploratory plots for analyzing taxonomic and functional profiles. | [(Parks et al., 2014)](https://paperpile.com/c/LXYLtZ/vtkGW) |
| UPARSE | An optimized pipeline for constructing OTUs de novo from next-generation sequencing reads. | [(Edgar, 2013)](https://paperpile.com/c/LXYLtZ/RbYD) |
| Vegan, R | Community Ecology R package. | [(Dixon and Philip, 2003)](https://paperpile.com/c/LXYLtZ/BYFTS) |
| XIPE-TOTEC | A statistical method to compare curated subsystems, to predict the physiology, metabolism, and ecology from metagenomes. | [(Rodriguez-Brito et al., 2006)](https://paperpile.com/c/LXYLtZ/RstsZ) |

[Afiahayati, Sato, K., and Sakakibara, Y. (2015). MetaVelvet-SL: an extension of the Velvet assembler to a de novo metagenomic assembler utilizing supervised learning. *DNA Res.* 22, 69–77.](http://paperpile.com/b/LXYLtZ/Rajw)

[Aggio, R. B. M. (2014). “Pathway Activity Profiling (PAPi): A Tool for Metabolic Pathway Analysis,” in *Methods in Molecular Biology*, 233–250.](http://paperpile.com/b/LXYLtZ/DVx2)

[Aggio, R., Villas-Boas, S. G., and Ruggiero, K. (2011). Metab: an R package for high-throughput analysis of metabolomics data generated by GC-MS. *Bioinformatics* 27, 2316–2318.](http://paperpile.com/b/LXYLtZ/yUkVH)

[Albanese, D., Fontana, P., De Filippo, C., Cavalieri, D., and Donati, C. (2015). MICCA: a complete and accurate software for taxonomic profiling of metagenomic data. *Sci. Rep.* 5, 9743.](http://paperpile.com/b/LXYLtZ/evtsa)

[Bankevich, A., Anton, B., Sergey, N., Dmitry, A., Gurevich, A. A., Mikhail, D., et al. (2012). SPAdes: A New Genome Assembly Algorithm and Its Applications to Single-Cell Sequencing. *J. Comput. Biol.* 19, 455–477.](http://paperpile.com/b/LXYLtZ/Mlin)

[Brady, A., and Salzberg, S. (2011). PhymmBL expanded: confidence scores, custom databases, parallelization and more. *Nat. Methods* 8, 367.](http://paperpile.com/b/LXYLtZ/mUo3)

[Brady, A., and Salzberg, S. L. (2009). Phymm and PhymmBL: metagenomic phylogenetic classification with interpolated Markov models. *Nat. Methods* 6, 673–676.](http://paperpile.com/b/LXYLtZ/rBsm)

[Cai, Y., and Sun, Y. (2011). ESPRIT-Tree: hierarchical clustering analysis of millions of 16S rRNA pyrosequences in quasilinear computational time. *Nucleic Acids Res.* 39, e95.](http://paperpile.com/b/LXYLtZ/WUX2T)

[Caporaso, J. G., Gregory Caporaso, J., Justin, K., Jesse, S., Kyle, B., Bushman, F. D., et al. (2010). QIIME allows analysis of high-throughput community sequencing data. *Nat. Methods* 7, 335–336.](http://paperpile.com/b/LXYLtZ/GARqO)

[Chen, S.-Y., Deng, F., Huang, Y., Jia, X., Liu, Y.-P., and Lai, S.-J. (2016). bioOTU: An Improved Method for Simultaneous Taxonomic Assignments and Operational Taxonomic Units Clustering of 16s rRNA Gene Sequences. *J. Comput. Biol.* 23, 229–238.](http://paperpile.com/b/LXYLtZ/RUu0)

[Costa, C., Maraschin, M., and Rocha, M. (2016). An R package for the integrated analysis of metabolomics and spectral data. *Comput. Methods Programs Biomed.* doi:](http://paperpile.com/b/LXYLtZ/9hsX)[10.1016/j.cmpb.2016.01.008](http://dx.doi.org/10.1016/j.cmpb.2016.01.008)[.](http://paperpile.com/b/LXYLtZ/9hsX)

[DeSantis, T. Z., Hugenholtz, P., Larsen, N., Rojas, M., Brodie, E. L., Keller, K., et al. (2006). Greengenes, a Chimera-Checked 16S rRNA Gene Database and Workbench Compatible with ARB. *Appl. Environ. Microbiol.* 72, 5069–5072.](http://paperpile.com/b/LXYLtZ/AVnf)

[Dixon, P., and Philip, D. (2003). VEGAN, a package of R functions for community ecology. *J. Veg. Sci.* 14, 927–930.](http://paperpile.com/b/LXYLtZ/BYFTS)

[Edgar, R. C. (2013). UPARSE: highly accurate OTU sequences from microbial amplicon reads. *Nat. Methods* 10, 996–998.](http://paperpile.com/b/LXYLtZ/RbYD)

[Fernandez-Albert, F., Llorach, R., Andres-Lacueva, C., and Perera, A. (2014). An R package to analyse LC/MS metabolomic data: MAIT (Metabolite Automatic Identification Toolkit). *Bioinformatics* 30, 1937–1939.](http://paperpile.com/b/LXYLtZ/LdvEZ)

[Finn, R. D., Clements, J., and Eddy, S. R. (2011). HMMER web server: interactive sequence similarity searching. *Nucleic Acids Res.* 39, W29–37.](http://paperpile.com/b/LXYLtZ/WsXx)

[Fu, L., Niu, B., Zhu, Z., Wu, S., and Li, W. (2012). CD-HIT: accelerated for clustering the next-generation sequencing data. *Bioinformatics* 28, 3150–3152.](http://paperpile.com/b/LXYLtZ/bvO2)

[Ghodsi, M., Liu, B., and Pop, M. (2011). DNACLUST: accurate and efficient clustering of phylogenetic marker genes. *BMC Bioinformatics* 12, 271.](http://paperpile.com/b/LXYLtZ/V6yw)

[Goll, J., Rusch, D. B., Tanenbaum, D. M., Thiagarajan, M., Li, K., Methé, B. A., et al. (2010). METAREP: JCVI metagenomics reports--an open source tool for high-performance comparative metagenomics. *Bioinformatics* 26, 2631–2632.](http://paperpile.com/b/LXYLtZ/uY3Lj)

[Haider, B., Ahn, T.-H., Bushnell, B., Chai, J., Copeland, A., and Pan, C. (2014). Omega: an overlap-graph de novo assembler for metagenomics. *Bioinformatics* 30, 2717–2722.](http://paperpile.com/b/LXYLtZ/0dWR)

[Heazlewood, J. L., Schrimpf, S. P., Dörte, B., Katrin, R., Andreas, T., and Emøke, B. (2015). Multi-Organism Proteomes (iMOP): Advancing our Understanding of Human Biology. *Proteomics* 15, 2885–2894.](http://paperpile.com/b/LXYLtZ/NDEsi)

[Hoff, K. J., Tech, M., Lingner, T., Daniel, R., Morgenstern, B., and Meinicke, P. (2008). Gene prediction in metagenomic fragments: a large scale machine learning approach. *BMC Bioinformatics* 9, 217.](http://paperpile.com/b/LXYLtZ/p5rO)

[Huson, D. H., and Weber, N. (2013). Microbial community analysis using MEGAN. *Methods Enzymol.* 531, 465–485.](http://paperpile.com/b/LXYLtZ/JDCV)

[Hwang, K., Oh, J., Kim, T.-K., Kim, B. K., Yu, D. S., Hou, B. K., et al. (2013). CLUSTOM: a novel method for clustering 16S rRNA next generation sequences by overlap minimization. *PLoS One* 8, e62623.](http://paperpile.com/b/LXYLtZ/PQaw)

[Kanehisa, M., Sato, Y., and Morishima, K. (2016). BlastKOALA and GhostKOALA: KEGG Tools for Functional Characterization of Genome and Metagenome Sequences. *J. Mol. Biol.* 428, 726–731.](http://paperpile.com/b/LXYLtZ/4SBJb)

[Katajamaa, M., Miettinen, J., and Oresic, M. (2006). MZmine: toolbox for processing and visualization of mass spectrometry based molecular profile data. *Bioinformatics* 22, 634–636.](http://paperpile.com/b/LXYLtZ/hgBGx)

[Keegan, K. P., Glass, E. M., and Meyer, F. (2016). MG-RAST, a Metagenomics Service for Analysis of Microbial Community Structure and Function. *Methods Mol. Biol.* 1399, 207–233.](http://paperpile.com/b/LXYLtZ/FQipi)

[Kelley, D. R., Liu, B., Delcher, A. L., Pop, M., and Salzberg, S. L. (2012). Gene prediction with Glimmer for metagenomic sequences augmented by classification and clustering. *Nucleic Acids Res.* 40, e9.](http://paperpile.com/b/LXYLtZ/CyffP)

[Koparde, V. N., Adkins, R. S., Fettweis, J. M., Serrano, M. G., Buck, G. A. A., Reimers, M. A., et al. (2014). BOTUX: bayesian-like operational taxonomic unit examiner. *Int. J. Comput. Biol. Drug Des.* 7, 130–145.](http://paperpile.com/b/LXYLtZ/IJKS)

[Kopylova, E., Noé, L., and Touzet, H. (2012). SortMeRNA: fast and accurate filtering of ribosomal RNAs in metatranscriptomic data. *Bioinformatics* 28, 3211–3217.](http://paperpile.com/b/LXYLtZ/7Dfg)

[Kristiansson, E., Hugenholtz, P., and Dalevi, D. (2009). ShotgunFunctionalizeR: an R-package for functional comparison of metagenomes. *Bioinformatics* 25, 2737–2738.](http://paperpile.com/b/LXYLtZ/2e9TW)

[Kultima, J. R., Coelho, L. P., Forslund, K., Huerta-Cepas, J., Li, S. S., Driessen, M., et al. (2016). MOCAT2: a metagenomic assembly, annotation and profiling framework. *Bioinformatics* 32, 2520–2523.](http://paperpile.com/b/LXYLtZ/sTrv)

[Langille, M. G. I., Zaneveld, J., Caporaso, J. G., McDonald, D., Knights, D., Reyes, J. A., et al. (2013). Predictive functional profiling of microbial communities using 16S rRNA marker gene sequences. *Nat. Biotechnol.* 31, 814–821.](http://paperpile.com/b/LXYLtZ/ZcJsW)

[Lee, A. Y., Lee, C. S., and Van Gelder, R. N. (2016). Scalable metagenomics alignment research tool (SMART): a scalable, rapid, and complete search heuristic for the classification of metagenomic sequences from complex sequence populations. *BMC Bioinformatics* 17, 292.](http://paperpile.com/b/LXYLtZ/8uCb)

[Leung, H. C. M., Siu-Ming, Y., John, P., and Chin, F. Y. L. (2013). IDBA-MT: De Novo Assembler for Metatranscriptomic Data Generated from Next-Generation Sequencing Technology. *J. Comput. Biol.* 20, 540–550.](http://paperpile.com/b/LXYLtZ/vBRe)

[Li, D., Liu, C.-M., Luo, R., Sadakane, K., and Lam, T.-W. (2015). MEGAHIT: an ultra-fast single-node solution for large and complex metagenomics assembly via succinct de Bruijn graph. *Bioinformatics* 31, 1674–1676.](http://paperpile.com/b/LXYLtZ/Smihm)

[Li, D., Luo, R., Liu, C.-M., Leung, C.-M., Ting, H.-F., Sadakane, K., et al. (2016). MEGAHIT v1.0: A fast and scalable metagenome assembler driven by advanced methodologies and community practices. *Methods*. doi:](http://paperpile.com/b/LXYLtZ/JSYae)[10.1016/j.ymeth.2016.02.020](http://dx.doi.org/10.1016/j.ymeth.2016.02.020)[.](http://paperpile.com/b/LXYLtZ/JSYae)

[Liu, Y., Guo, J., Hu, G., and Zhu, H. (2013a). Gene prediction in metagenomic fragments based on the SVM algorithm. *BMC Bioinformatics* 14 Suppl 5, S12.](http://paperpile.com/b/LXYLtZ/cQrA)

[Liu, Y., Guo, J., Hu, G., and Zhu, H. (2013b). Gene prediction in metagenomic fragments based on the SVM algorithm. *BMC Bioinformatics* 14 Suppl 5, S12.](http://paperpile.com/b/LXYLtZ/2VqQ)

[Li, W., and Godzik, A. (2006). Cd-hit: a fast program for clustering and comparing large sets of protein or nucleotide sequences. *Bioinformatics* 22, 1658–1659.](http://paperpile.com/b/LXYLtZ/rYDP)

[Loman, N. J., and Quinlan, A. R. (2014). Poretools: a toolkit for analyzing nanopore sequence data. *Bioinformatics* 30, 3399–3401.](http://paperpile.com/b/LXYLtZ/L1uY3)

[Manor, O., and Borenstein, E. (2015). MUSiCC: a marker genes based framework for metagenomic normalization and accurate profiling of gene abundances in the microbiome. *Genome Biol.* 16, 53.](http://paperpile.com/b/LXYLtZ/Ymad)

[Markowitz, V. M., Chen, I.-M. A., Chu, K., Szeto, E., Palaniappan, K., Pillay, M., et al. (2014). IMG/M 4 version of the integrated metagenome comparative analysis system. *Nucleic Acids Res.* 42, D568–73.](http://paperpile.com/b/LXYLtZ/OZGvW)

[Markowitz, V. M., Ivanova, N., Palaniappan, K., Szeto, E., Korzeniewski, F., Lykidis, A., et al. (2006). An experimental metagenome data management and analysis system. *Bioinformatics* 22, e359–67.](http://paperpile.com/b/LXYLtZ/qUgYg)

[Martinez, X., Pozuelo, M., Pascal, V., Campos, D., Gut, I., Gut, M., et al. (2016). MetaTrans: an open-source pipeline for metatranscriptomics. *Sci. Rep.* 6, 26447.](http://paperpile.com/b/LXYLtZ/MXQa7)

[McMurdie, P. J., and Holmes, S. (2014). Shiny-phyloseq: Web application for interactive microbiome analysis with provenance tracking. *Bioinformatics* 31, 282–283.](http://paperpile.com/b/LXYLtZ/gq2zs)

[McMurdie, P. J., and Susan, H. (2013). phyloseq: An R Package for Reproducible Interactive Analysis and Graphics of Microbiome Census Data. *PLoS One* 8, e61217.](http://paperpile.com/b/LXYLtZ/mtYe0)

[Mikheenko, A., Saveliev, V., and Gurevich, A. (2016). MetaQUAST: evaluation of metagenome assemblies. *Bioinformatics* 32, 1088–1090.](http://paperpile.com/b/LXYLtZ/KNJwk)

[Namiki, T., Hachiya, T., Tanaka, H., and Sakakibara, Y. (2012). MetaVelvet: an extension of Velvet assembler to de novo metagenome assembly from short sequence reads. *Nucleic Acids Res.* 40, e155–e155.](http://paperpile.com/b/LXYLtZ/tp0l)

[Navas-Molina, J. A., Peralta-Sánchez, J. M., González, A., McMurdie, P. J., Vázquez-Baeza, Y., Xu, Z., et al. (2013). Advancing our understanding of the human microbiome using QIIME. *Methods Enzymol.* 531, 371–444.](http://paperpile.com/b/LXYLtZ/lAUam)

[Ni, Y., Yueqiong, N., Jun, L., and Gianni, P. (2016). COMAN: a web server for comprehensive metatranscriptomics analysis. *BMC Genomics* 17. doi:](http://paperpile.com/b/LXYLtZ/tr3i)[10.1186/s12864-016-2964-z](http://dx.doi.org/10.1186/s12864-016-2964-z)[.](http://paperpile.com/b/LXYLtZ/tr3i)

[Nodzenski, M., Muehlbauer, M. J., Bain, J. R., Reisetter, A. C., Lowe, W. L., Jr, and Scholtens, D. M. (2014). Metabomxtr: an R package for mixture-model analysis of non-targeted metabolomics data. *Bioinformatics* 30, 3287–3288.](http://paperpile.com/b/LXYLtZ/ug2r1)

[Noecker, C., Eng, A., Srinivasan, S., Theriot, C. M., Young, V. B., Jansson, J. K., et al. (2016). Metabolic Model-Based Integration of Microbiome Taxonomic and Metabolomic Profiles Elucidates Mechanistic Links between Ecological and Metabolic Variation. *mSystems* 1. doi:](http://paperpile.com/b/LXYLtZ/Q2IH)[10.1128/mSystems.00013-15](http://dx.doi.org/10.1128/mSystems.00013-15)[.](http://paperpile.com/b/LXYLtZ/Q2IH)

[Ogata, H., Goto, S., Sato, K., Fujibuchi, W., Bono, H., and Kanehisa, M. (1999). KEGG: Kyoto Encyclopedia of Genes and Genomes. *Nucleic Acids Res.* 27, 29–34.](http://paperpile.com/b/LXYLtZ/06qpe)

[Ounit, R., Wanamaker, S., Close, T. J., and Lonardi, S. (2015). CLARK: fast and accurate classification of metagenomic and genomic sequences using discriminative k-mers. *BMC Genomics* 16, 236.](http://paperpile.com/b/LXYLtZ/feaW)

[Parks, D. H., Tyson, G. W., Hugenholtz, P., and Beiko, R. G. (2014). STAMP: statistical analysis of taxonomic and functional profiles. *Bioinformatics* 30, 3123–3124.](http://paperpile.com/b/LXYLtZ/vtkGW)

[Peng, Y., Leung, H. C. M., Yiu, S. M., and Chin, F. Y. L. (2012). IDBA-UD: a de novo assembler for single-cell and metagenomic sequencing data with highly uneven depth. *Bioinformatics* 28, 1420–1428.](http://paperpile.com/b/LXYLtZ/FNBV)

[Quast, C., Pruesse, E., Yilmaz, P., Gerken, J., Schweer, T., Yarza, P., et al. (2013). The SILVA ribosomal RNA gene database project: improved data processing and web-based tools. *Nucleic Acids Res.* 41, D590–6.](http://paperpile.com/b/LXYLtZ/Etemh)

[Rho, M., Tang, H., and Ye, Y. (2010). FragGeneScan: predicting genes in short and error-prone reads. *Nucleic Acids Res.* 38, e191–e191.](http://paperpile.com/b/LXYLtZ/9vF0W)

[Rodriguez-Brito, B., Rohwer, F., and Edwards, R. A. (2006). An application of statistics to comparative metagenomics. *BMC Bioinformatics* 7, 162.](http://paperpile.com/b/LXYLtZ/RstsZ)

[Schloss, P. D., Westcott, S. L., Ryabin, T., Hall, J. R., Hartmann, M., Hollister, E. B., et al. (2009). Introducing mothur: Open-Source, Platform-Independent, Community-Supported Software for Describing and Comparing Microbial Communities. *Appl. Environ. Microbiol.* 75, 7537–7541.](http://paperpile.com/b/LXYLtZ/L3cF)

[Segata, N., Waldron, L., Ballarini, A., Narasimhan, V., Jousson, O., and Huttenhower, C. (2012). Metagenomic microbial community profiling using unique clade-specific marker genes. *Nat. Methods* 9, 811–814.](http://paperpile.com/b/LXYLtZ/AHbV)

[Simpson, J. T., and Durbin, R. (2012). Efficient de novo assembly of large genomes using compressed data structures. *Genome Res.* 22, 549–556.](http://paperpile.com/b/LXYLtZ/lCH6)

[Simpson, J. T., Wong, K., Jackman, S. D., Schein, J. E., Jones, S. J. M., and Birol, I. (2009). ABySS: a parallel assembler for short read sequence data. *Genome Res.* 19, 1117–1123.](http://paperpile.com/b/LXYLtZ/KvZo)

[Sunagawa, S., Mende, D. R., Zeller, G., Izquierdo-Carrasco, F., Berger, S. A., Kultima, J. R., et al. (2013). Metagenomic species profiling using universal phylogenetic marker genes. *Nat. Methods* 10, 1196–1199.](http://paperpile.com/b/LXYLtZ/7PO2)

[Treangen, T. J., Koren, S., Sommer, D. D., Liu, B., Astrovskaya, I., Ondov, B., et al. (2013). MetAMOS: a modular and open source metagenomic assembly and analysis pipeline. *Genome Biol.* 14, R2.](http://paperpile.com/b/LXYLtZ/dIlF)

[Wang, X., Xiaoyu, W., Jin, Y., Yijun, S., and Volker, M. (2013). M-pick, a modularity-based method for OTU picking of 16S rRNA sequences. *BMC Bioinformatics* 14, 43.](http://paperpile.com/b/LXYLtZ/Fdqb)

[Watson, M., Thomson, M., Risse, J., Santoyo-Lopez, J., Talbot, R., Gharbi, K., et al. (2014). poRe: an R package for the visualization and analysis of nanopore sequencing data. doi:](http://paperpile.com/b/LXYLtZ/jyNb2)[10.1101/007567](http://dx.doi.org/10.1101/007567)[.](http://paperpile.com/b/LXYLtZ/jyNb2)

[Wehrens, R., Weingart, G., and Mattivi, F. (2014). metaMS: an open-source pipeline for GC-MS-based untargeted metabolomics. *J. Chromatogr. B Analyt. Technol. Biomed. Life Sci.* 966, 109–116.](http://paperpile.com/b/LXYLtZ/956vS)

[Wei, Z.-G., and Zhang, S.-W. (2015). MtHc: a motif-based hierarchical method for clustering massive 16S rRNA sequences into OTUs. *Mol. Biosyst.* 11, 1907–1913.](http://paperpile.com/b/LXYLtZ/tQb4)

[Wilke, A., Bischof, J., Gerlach, W., Glass, E., Harrison, T., Keegan, K. P., et al. (2016). The MG-RAST metagenomics database and portal in 2015. *Nucleic Acids Res.* 44, D590–4.](http://paperpile.com/b/LXYLtZ/BEJlQ)

[Wishart, D. S., Jewison, T., Guo, A. C., Wilson, M., Knox, C., Liu, Y., et al. (2012). HMDB 3.0--The Human Metabolome Database in 2013. *Nucleic Acids Res.* 41, D801–D807.](http://paperpile.com/b/LXYLtZ/eBNQ)

[Xia, J., Psychogios, N., Young, N., and Wishart, D. S. (2009). MetaboAnalyst: a web server for metabolomic data analysis and interpretation. *Nucleic Acids Res.* 37, W652–60.](http://paperpile.com/b/LXYLtZ/yCzi)

[Ye, Y., and Tang, H. (2009). An ORFome assembly approach to metagenomics sequences analysis. *J. Bioinform. Comput. Biol.* 7, 455–471.](http://paperpile.com/b/LXYLtZ/WmgP)

[Zhang, Y., Ji, P., Wang, J., and Zhao, F. (2016). RiboFR-Seq: a novel approach to linking 16S rRNA amplicon profiles to metagenomes. *Nucleic Acids Res.* doi:](http://paperpile.com/b/LXYLtZ/4KLfP)[10.1093/nar/gkw165](http://dx.doi.org/10.1093/nar/gkw165)[.](http://paperpile.com/b/LXYLtZ/4KLfP)

[Zimin, A. V., Marçais, G., Puiu, D., Roberts, M., Salzberg, S. L., and Yorke, J. A. (2013). The MaSuRCA genome assembler. *Bioinformatics* 29, 2669–2677.](http://paperpile.com/b/LXYLtZ/peQb)
